# Coumarin Glycosides Reverse Enterococci-Facilitated Enteric Infections

**DOI:** 10.34133/research.0374

**Published:** 2024-05-16

**Authors:** Wenjiao Xu, Guixin Yuan, Yuwen Fang, Xiaojia Liu, Xiaowei Ma, Kui Zhu

**Affiliations:** ^1^ National Key Laboratory of Veterinary Public Health and Safety, College of Veterinary Medicine, China Agricultural University, Beijing 100193, China.; ^2^ Engineering Research Center of Animal Innovative Drugs and Safety Evaluation, Ministry of Education, College of Veterinary Medicine, China Agricultural University, Beijing 100193, China.; ^3^ Ministry of Agriculture and Rural Affairs Key Laboratory for Crop Pest Monitoring and Green Control, China Agricultural University, Beijing 100193, China.

## Abstract

Commensal enterococci with pathogenic potential often facilitate the growth of diverse pathogens, thereby exacerbating infections. However, there are few effective therapeutic strategies to prevent and intervene in enterococci-mediated polymicrobial infections. Here, we find that enterococci at high density drive the expansion and pathogenicity of enteric *Salmonella enterica* serotype Typhimurium (*S*. Tm). Subsequently, we show that the driving role of enterococci in such infections is counteracted by dietary coumarin glycosides in vivo. Enterococci, which are tolerant of iron-deficient environments, produce β-glucosidases to hydrolyze coumarin glycosides into bioactive aglycones, inhibiting *S*. Tm growth and ameliorating the severity of *S*. Tm-induced symptoms by inducing iron limitation. Overall, we demonstrate that coumarin glycosides as a common diet effectively reverse enterococci-facilitated enteric infections, providing an alternative intervention to combat polymicrobial infections.

## Introduction

Enterococci are commensals of the gut microbiota in both animals and humans, causing latent risks under specific conditions and therefore considered as “pathobionts”. Many factors can favor the overgrowth of enterococci in the gut, including medical reasons, dietary habits, and susceptible individuals [1]. Enterococcal intestinal domination, especially the devastating dissemination of vancomycin-resistant enterococci, has been identified as an indicator of subsequent infections and adverse outcomes [2,3]. Numerous studies demonstrated that enterococci facilitate the growth and pathogenicity of various pathogens, such as *Clostridioides difficile*, *Staphylococcus aureus*, *Pseudomonas aeruginosa*, *Escherichia coli*, *Gardnerella vaginalis*, and *Candida albicans*, via up-regulation of virulence, metabolic cross-feeding, biofilm formation, immune evasion, and transmission of drug resistance [4–9]. For instance, enterococci can promote toxin production of *C. difficile* through arginine catabolism in the gut, enhancing the fitness of *C. difficile* [10]. The driving role of enterococci in polymicrobial infection contributes to recurrent infections with elevated rates of treatment failure [11].

Given that multidrug-resistant enterococci are prone to overgrow in the gut after antibiotic treatments [12,13], enterococci-associated polymicrobial infections are difficult to eradicate by routine antibiotics. Seriously, the ability of enterococci to promote the proliferation, pathogenesis, and persistence of various pathogens further compromises the efficacy of existing antibiotics [14,15]. As an example, *Enterococcus faecalis* fuels the rapid autoaggregation and growth of *E. coli*, thus reinforcing dual-species biofilms to withstand harsh stresses including antibiotic treatments [16]. Consequently, exploring effective therapeutic strategies of enterococci-associated polymicrobial infections is an emergent and challenging problem to be solved.

Mounting evidence suggests that dietary plants offer a promising approach to improving host health and preventing diseases [17]. Natural coumarins, ubiquitously distributed in plant-based foods and medicinal herbs, possess the advances in structural diversity, accessibility, and robust antimicrobial activity, with an average intake of 1.2 mg/d for a 60-kg consumer [18,19]. However, many natural products, including coumarins, are often glycoconjugated as glycosides to enable storage and solubility in plants, exhibiting less or no antimicrobial activity compared with the deglycosylated counterparts (aglycones) [20,21]. Therefore, the deglycosylation of coumarin glycosides is the limiting step to modulate their bioavailability and bioactivities in hosts [22]. Although pioneering studies have reported that dietary glycosides can be converted into bioactive aglycones by gut microorganisms [23], the specific bacteria and proteins involved in coumarin glycoside utilization remain poorly understood.

In this work, we investigated the hydrolytic capacity of enterococci to coumarin glycosides, offering an effective approach to combating enterococci-mediated polymicrobial infections. First, we show the bacterial cooperation that enterococci promote the expansion and pathogenicity of enteri*c Salmonella enterica* serotype Typhimurium (*S*. Tm). Next, our findings illustrate that the oral administration of coumarin glycosides reverses the promotion of enterococci to *S*. Tm infection. Specifically, enterococci-derived β-glucosidases (BGLs) hydrolyze coumarin glycosides into antibacterial aglycones, inhibiting the growth of *S*. Tm by outcompeting for iron. The interventions of coumarin glycosides in enterococci-mediated polymicrobial infection are validated in both in vitro and animal models, suggesting that diet-based strategies are encouraging approaches to mitigating enteric polymicrobial infections.

## Results

### Enterococci promote the expansion and pathogenicity of *S*. Tm

To identify the driving impact of enterococci on polymicrobial infections in the gut, we used *Enterococcus faecium* and enteropathogenic *S*. Tm as a model. First, we quantified their interactions by assessing the growth dynamics of *S*. Tm 15E475 in cocultures with *E. faecium* CAU369 at inoculum ratios of 1:1, 1:10, and 1:100 (*S*. Tm: *E. faecium*), respectively. The growth of *S*. Tm in the cocultures presented an identical pattern to that of monocultures (Fig. S1A), indicating that *E. faecium* does not affect the growth of *S*. Tm under planktonic cultures. Given that enterococci-associated biofilm formation frequently enhances the proliferation of other pathogens, we determined whether the interaction between *E. faecium* CAU369 and *S*. Tm 15E475 affects biofilm biomass based on the standard crystal violet assay. We found that the cocultures showed significantly higher biofilm formation than monocultures at 72 and 96 h (Fig. S1B), suggesting that the promotion of enterococci to *S*. Tm growth is dependent on local bacterial communication.

**Fig. 1.**
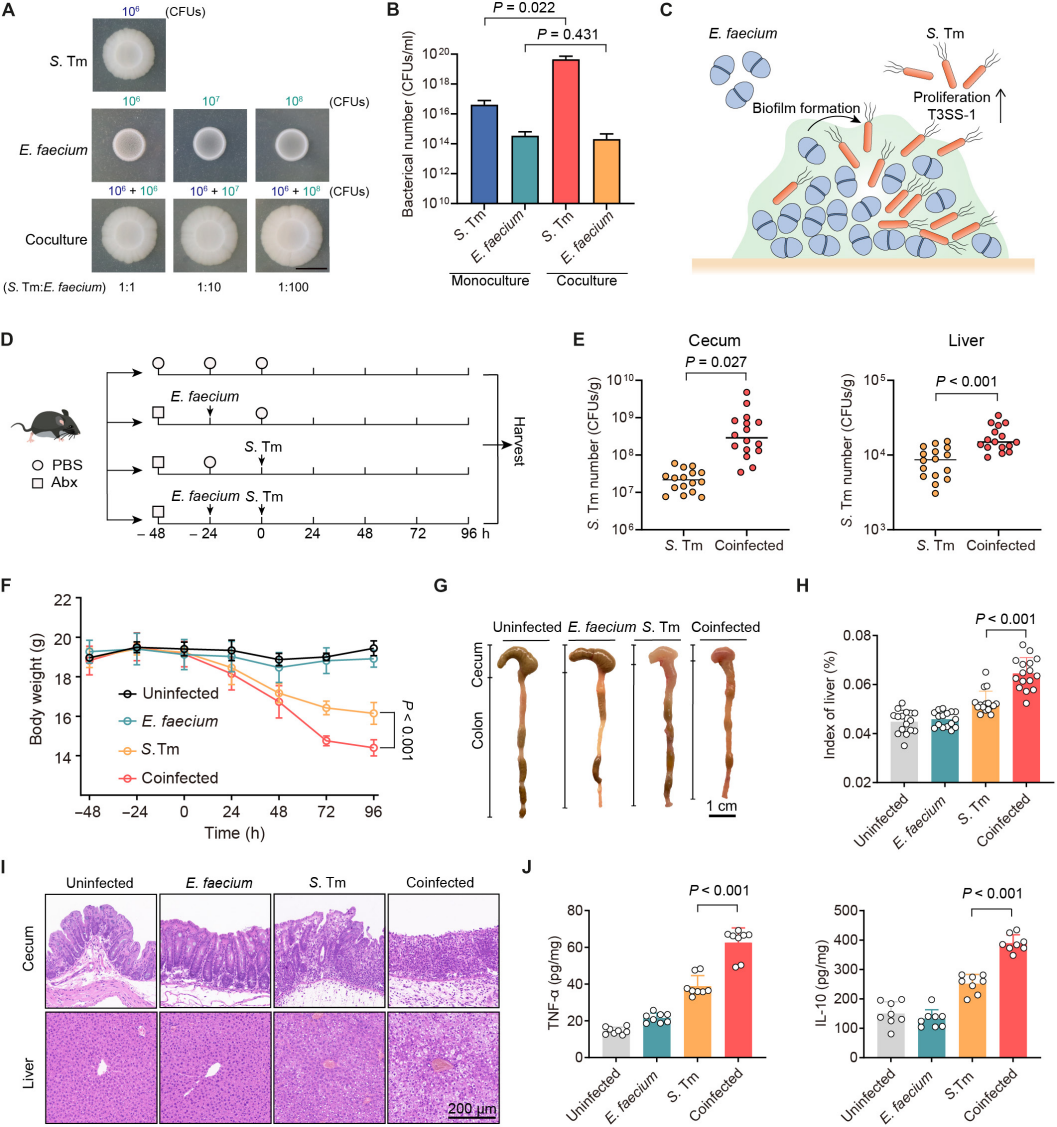
Enterococci promote the expansion and pathogenicity of *S*. Tm. (A) Representative macrocolony images of *S*. Tm 15E475, *E. faecium* CAU369, and cocultures (1:1, 1:10, and 1:100) on agar plates. The starting inoculum of *S*. Tm is 10^6^ CFUs in all macrocolonies. Scale bar, 1 cm. (B) Bacterial counts of *S*. Tm 15E475 and *E. faecium* CAU369 in mono- or cocultured (1:100) macrocolonies. *n* = 3 biological replicates with 3 technical replicates. (C) Schematic diagram of *E. faecium* enhanced growth and virulence of *S*. Tm within biofilms. (D) Experimental scheme. In the coinfected group, antibiotic-treated (Abx) mice were orally precolonized with *E. faecium* CAU369 before challenge with *S*. Tm 15E475. Mice were colonized with *E. faecium* CAU369 or *S*. Tm 15E475 alone as the mono-infected control. *n* = 16 per group from 2 independent experiments. (E) Enumeration of *S*. Tm in cecal contents and livers at 96 h after *S*. Tm infection. (F) Body weight of uninfected, mono-infected, and coinfected mice. (G) Macroscopic phenotype of cecum and colon from corresponding treatments. (H) Organ indices of the liver. (I) Hematoxylin and eosin (H&E) staining images of the corresponding samples. (J) Levels of TNF-α and IL-10 in the cecum. Results represent the means ± SE. *P* values were calculated using one-way ANOVA with the LSD post hoc test (B) or independent-samples *t* test (E, F, H, and J).

To better mimic the environment of polymicrobial interactions, we used macrocolonies as a surrogate for biofilm formation tests to determine how enterococci modulate the growth of *S*. Tm. We grew the cocultured macrocolonies at various inoculum ratios of 1:1, 1:10, and 1:100 (*S*. Tm: *E. faecium*), maintaining the initial number of *S*. Tm constant. In comparison with macrocolonies of single species, we observed augmented and specific accumulation of biomass in cocultured macrocolonies (Fig. 1A). The higher ratio of *E. faecium* to *S*. Tm (from 1:1 to 1:100) potentiated the production of biofilm biomass in macrocolonies (Fig. 1B), indicating by the bacterial load that the presence of enterococci resulted in significantly more *S*. Tm number in cocultured macrocolonies (*P* = 0.022). Conversely, the number of *E. faecium* remained similar between single-and dual-species macrocolonies (*P* = 0.431), suggesting that *E. faecium* is still growing well when the cocultured *S*. Tm growth is robust. However, we did not observe a similar phenomenon in other dual-species macrocolonies, including methicillin-resistant *S*. *aureus* T144, *E*. *coli* B2 and *P*. *aeruginosa* PAO1 in the presence of *E. faecium* CAU369 (Fig. S1C). Consequently, we focused on the high inoculum ratio of *E. faecium* to *S*. Tm for subsequent studies.

Next, we investigated the impact of *E. faecium* on the virulence of invading *S*. Tm. Our findings revealed that high-density enterococci significantly up-regulated the relative expression of virulence genes (VGs) associated with T3SS-1 of *S*. Tm (Fig. S1D), which located in *Salmonella* pathogenicity islands-1, facilitating rapid replication of *S*. Tm in the gut [24,25]. Overall, these results indicate that *E. faecium* enhances the growth and virulence of *S*. Tm within biofilms (Fig. 1C). To evaluate the effect of *E. faecium* on *S*. Tm infection in vivo, we established a coinfected mouse model (Fig. 1D). Given that enterococcal overgrowth in the gut frequently serves as a precursor to subsequent infections of other pathogens [2], we gave mice an oral administration of *E. faecium* CAU369 24 h in advance to establish an ecological niche dominated by enterococci prior to *S*. Tm infection (Fig. S2A). Notably, mice coinfected with *E. faecium* showed higher burdens of *S*. Tm in cecal contents and livers compared with those infected with *S*. Tm alone (Fig. 1E). Meanwhile, coinfected mice showed less food intake and more weight loss (Fig. 1F and Fig. S2B). The intestines and livers of coinfected mice exhibited more severe injuries, as indicated by greater shrinkage and hemorrhage of the large intestine (Fig. 1G); a more swollen, yellowish, and brittle phenotype and increased organ indices of the liver (Fig. S2C and Fig. 1H); more integrity loss and inflammatory cell infiltration in the cecum, colon, and liver (Fig. 1I and Fig. S2D); and significantly up-regulated levels of inflammatory factor in the cecum and liver (Fig. 1J and Fig. S2E). Taken together, these findings demonstrate the successful establishment of a model wherein enterococci drive the growth of enteropathogenic bacteria, laying the groundwork for further exploration of therapeutic strategies.

### Coumarin glucosides inhibit *S*. Tm growth in the presence of enterococci

**Fig. 2.**
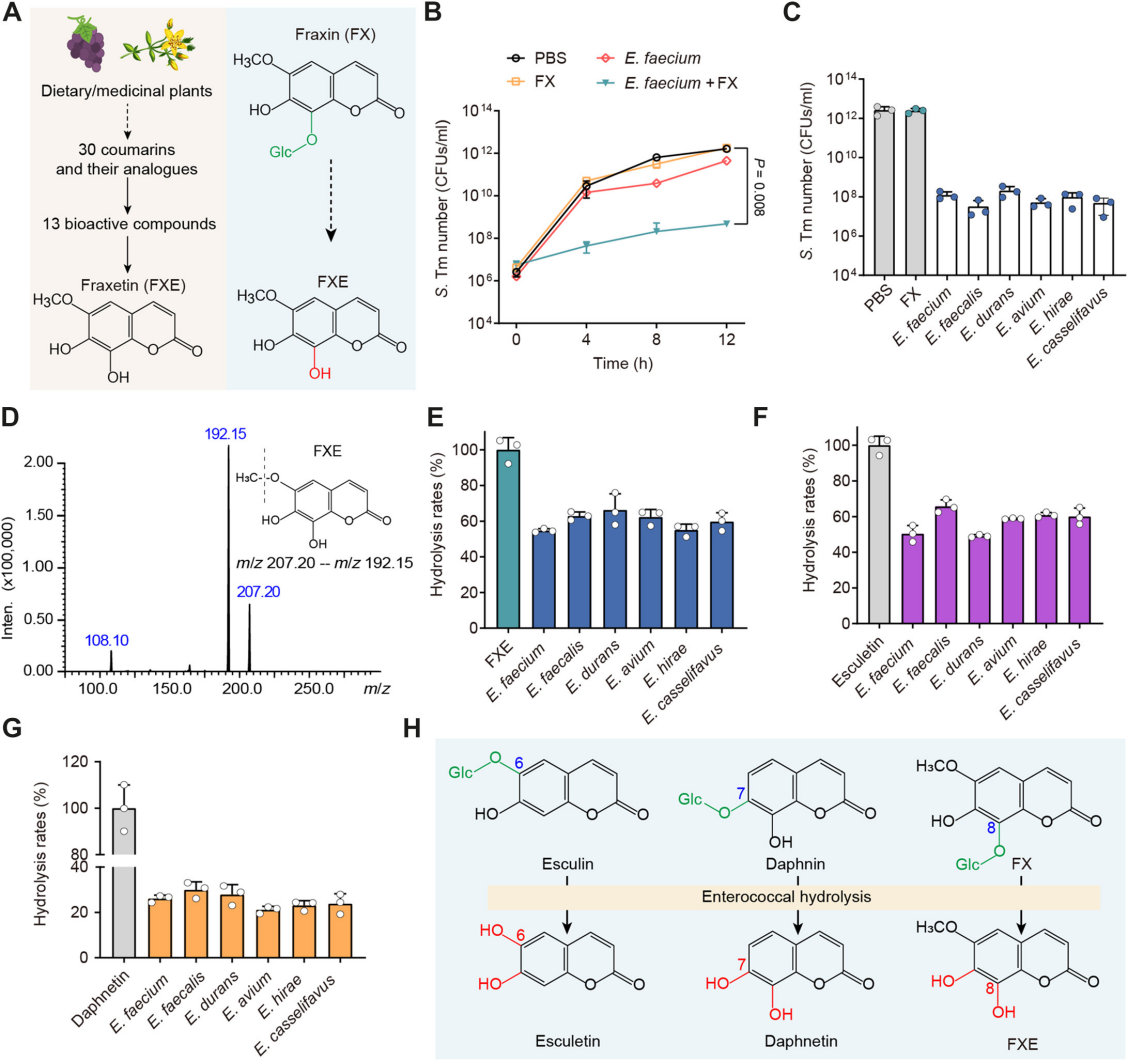
Coumarin glycosides inhibit *S*. Tm growth in the presence of enterococci. (A) Scheme representation of screening bioactive coumarin compounds. Glc, glucose. (B) Population dynamics of *S*. Tm 15E475 in the cocultures with *E. faecium* CAU369, FX, or *E. faecium* CAU369 plus FX for 12 h. (C) Enumeration of *S*. Tm 15E475 in cocultures with FX or diverse enterococcal species plus FX at 12 h. (D) The MS/MS spectrum and chemical structure of FXE. *m*/*z*, mass/charge ratio. (E to G) Hydrolytic rates of diverse enterococcal species to FX (E), esculin (F), and daphnetin (G) based on LC-MS/MS analysis. (H) Schematic diagram illustrating the hydrolysis of glycosidic bonds in dietary coumarin glycosides by enterococci. *n* = 3 biological replicates with 3 technical replicates. Results represent the means ± SE. *P* values were calculated using the independent-samples *t* test.

Coumarins, in the form of aglycones or glycosides, are abundantly present in the human diet [19], with their bioavailability dependent on structures and stability. Aglycones can be readily absorbed in the small intestine, whereas glycosides exhibit very low absorptivity and primarily transit to the large intestine [26,27]. Therefore, coumarin glycosides have the robust advantage of oral administration, allowing them to reach infection sites of enteropathogenic bacteria. To screen representative coumarin compounds, we collected 30 coumarins and their analogs from dietary and medicinal plants and assessed their antibacterial activities (Fig. 2A). Fraxetin (FXE) showed the predominant antibacterial activity (Table S1). Therefore, we selected FXE and its glycoside, fraxin (FX), as the representative aglycone and glycoside, respectively, for following mechanistic studies.

To define the role of enterococci in the biological activity of coumarin glycosides, we examined the growth kinetics of *S*. Tm in cocultures with *E. faecium* plus FX. Intriguingly, the presence of *E. faecium* with FX markedly inhibited the *S*. Tm growth, whereas the single addition of *E. faecium* or FX has no antibacterial activity (Fig. 2B and Fig. S3), implying that *E. faecium* may hydrolyze inactive FX into bioactive FXE. To ascertain the generality of this phenomenon among *E. faecium* isolates and other enterococcal species, we tested the *S*. Tm number in cocultures with diverse enterococcal isolates plus FX. All enterococcal isolates displayed similar activities that their presence with FX inhibited the growth of *S*. Tm (Fig. 2C and Fig. S4A). To directly verify the hydrolytic capacity of enterococci to FX, we constructed the tandem mass spectrometry (MS/MS) spectrum of FXE (Fig. 2D) and measured the concentrations of FXE in cocultured supernatants of FX with diverse enterococcal species using liquid chromatography with MS/MS (LC-MS/MS) analysis. As anticipated, enterococci efficiently hydrolyzed FX into FXE, with an average hydrolytic rate exceeding 54% for all assayed enterococcal isolates (Fig. 2E). The glycosidic bond of FX is located at the C-8 position. To evaluate the diversity of substrates catalyzed by enterococci, we assessed the hydrolytic activity of enterococci on esculin and daphnin, whose glycosidic bonds are at positions C-6 and C-7, respectively. Consistently, enterococcal isolates are capable of hydrolyzing esculin and daphnin as well, with average hydrolysis rates of 57.47% and 25.35%, respectively (Fig. 2F and G and Fig. S4B). Altogether, these findings denote that enterococci hydrolyze coumarin glycosides into antibacterial aglycones (Fig. 2H), thereby inhibiting the growth of *S*. Tm.

### Enterococci-derived BGLs hydrolyze coumarin glycosides into antibacterial aglycones

**Fig. 3.**
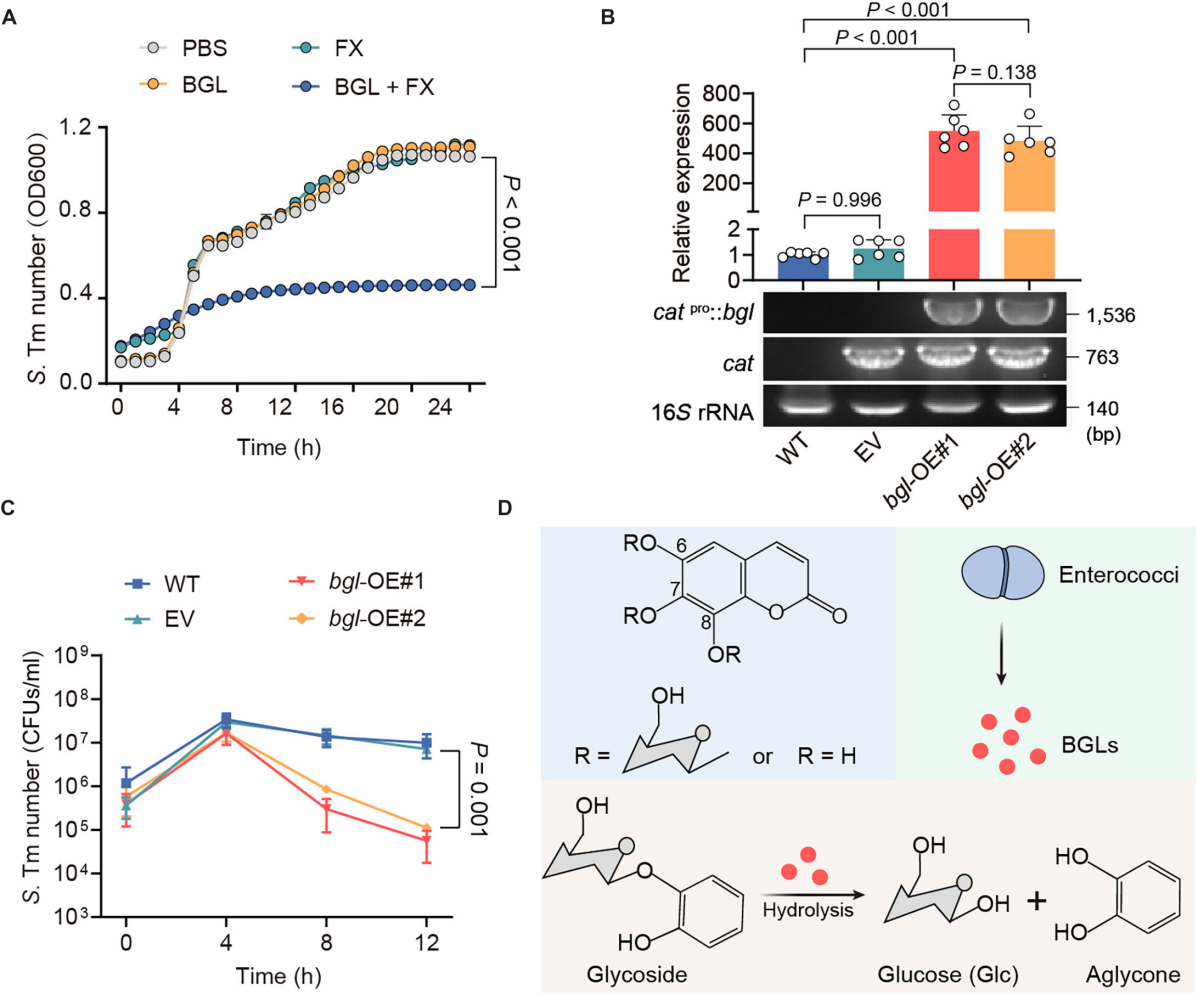
Enterococci-derived BGLs hydrolyze coumarin glycosides into antibacterial aglycones. (A) Growth curves of *S*. Tm 15E475 in cocultures with FX, BGL, or BGL plus FX for 24 h. OD600, optical density at 600 nm. (B) Relative expression of *bgl* in wild-type (WT; *E. faecalis* JH2-2), EV, and overexpression strains (*bgl*-OE#1 and *bgl*-OE#2). (C) Growth dynamics of *S*. Tm 15E475 in cocultures with FX plus wild-type, EV, *bgl*-OE#1, or *bgl*-OE#2, respectively. (D) Schematic representation of enterococci hydrolyzing glycosides to aglycones via secreting BGLs. *n* = 3 biological replicates with 3 technical replicates. Results represent the means ± SE. *P* values were calculated using one-way ANOVA with the LSD post hoc test (B) or independent-samples *t* test (C).

Considering that plant-derived BGLs are responsible for the hydrolysis of coumarin glycosides [28], we hypothesized that enterococci hydrolyze coumarin glycosides by secreting similar BGLs. First, we screened the *bgl* gene encoding BGLs in enterococcal isolates based on whole-genome sequencing. As expected, *E. faecium* isolates, including *E. faecium* CAU369, carry homogenous *bgl* genes (Fig. S5A). To purify such BGLs, we amplified the coding sequence (CDS) fragments of *bgl* from the cDNA of *E. faecium* CAU369 and cloned them into the pET-28a(+) vector. Subsequently, recombinant BGL proteins were expressed and characterized (Fig. S5B and C). To evaluate the activity of purified BGLs, we further measured *S*. Tm growth curves in cocultures with FX plus BGLs for 24 h. The growth of *S*. Tm was noticeably suppressed in cocultures with FX plus BGLs, compared with monocultures of FX (Fig. 3A). In addition, *bgl*-overexpression strains were constructed by transferring the *bgl* gene into the wild-type *E. faecalis* JH2-2 (Fig. S5D). We observed that the *bgl*-overexpression strains (*bgl*-OE#1 and *bgl*-OE#2) show higher expression levels of *bgl* and elevated hydrolytic capacity of FX (Fig. 3B and C). Taken together, these results indicate that enterococci produce BGLs to hydrolyze coumarin glycosides into antibacterial aglycones (Fig. 3D).

### Bioactive aglycones inhibit *S*. Tm growth by chelating iron(III)

The antibacterial activity of coumarin aglycones is closely associated with their hydroxyl groups [18], prompting us to perform structure–activity relationship analysis of compounds in Table S1. Compared to compounds with no- or monohydroxyl groups (16.7%, 2 of 12), those with 2 or more hydroxyl groups (61.1%, 11 of 18) predominated among active compounds. Notably, compounds with neighboring hydroxyl groups attached to the skeleton (catechol aglycones) exhibited strong antibacterial activity, accounting for 90.9% (10 of 11) of active compounds. These catechol aglycones share structural similarity with catecholate-type siderophores, which are known to acquire iron(III) for many bacteria [29]. We hypothesized that the antibacterial mechanism of catechol aglycones depends on iron chelation.

**Fig. 4.**
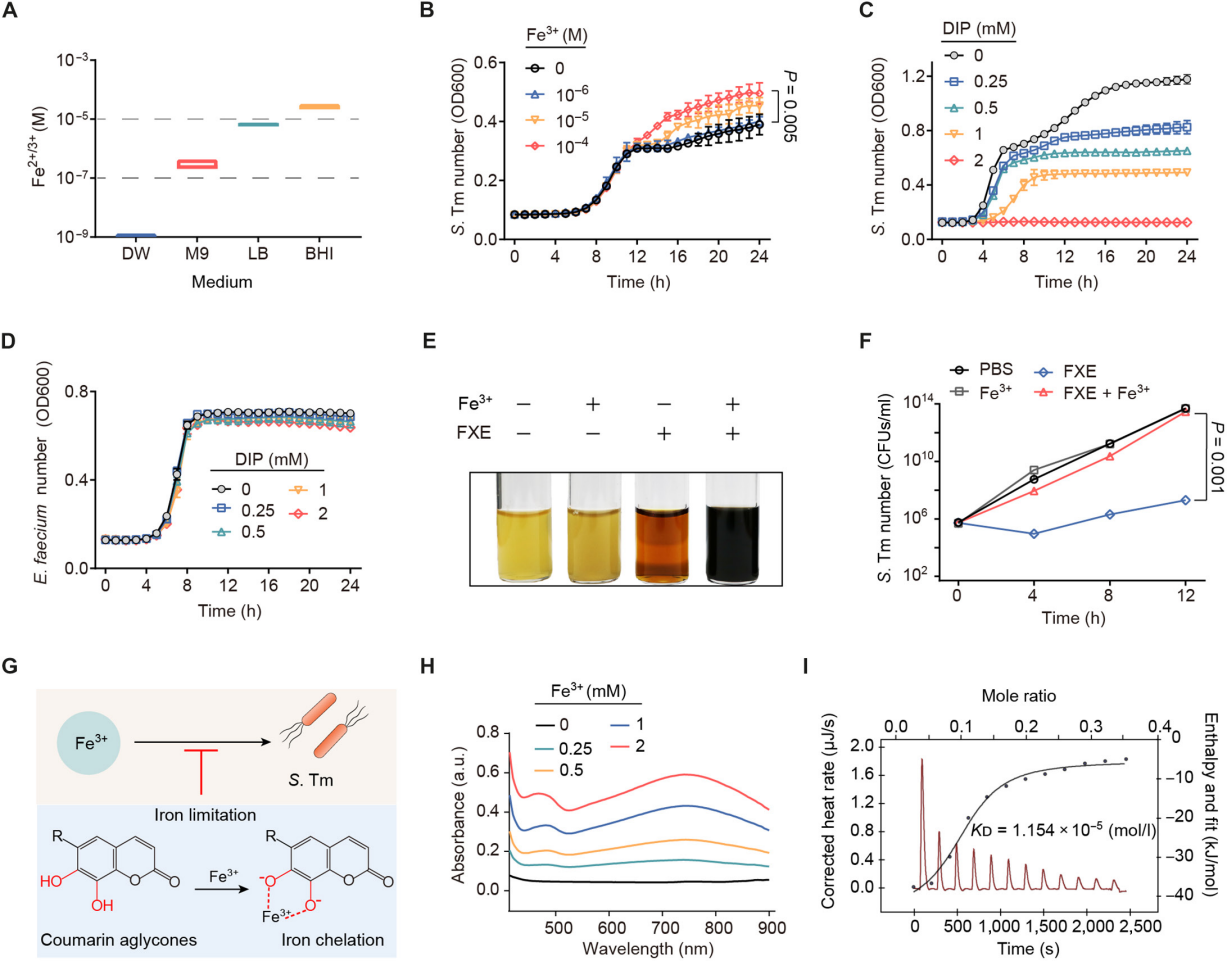
Coumarin aglycones inhibit *S*. Tm growth by chelating iron(III). (A) The concentration of iron in different media including distilled water (DW), M9, Lilly–Barnett (Lilly–Barnett), and BHI. (B) Growth curves of *S*. Tm 15E475 in M9 broth with the addition of iron(III) for 24 h. (C) Growth curves of *S*. Tm 15E475 with the addition of DIP for 24 h. (D) Growth curves of *E. faecium* CAU369 with the addition of DIP for 24 h. (E and F) The addition of excess iron(III) offsets the antibacterial activity of FXE. (E) The color change reaction of *S*. Tm 15E475 liquid cocultured with FXE, excess iron(III), or FXE plus excess iron(III), respectively. (F) The number of *S*. Tm 15E475 in the presence of FXE with excess iron(III). (G) Strategy scheme. Iron limitation caused by coumarin aglycones inhibits the growth of *S*. Tm. (H) The UV-Vis spectra of gradient concentrations of iron(III) with FXE (1 mM) in 5% DMSO buffer. a.u., arbitrary units. (I) Affinity of the chelation between iron(III) and FXE based on the ITC analysis. Iron(III) (2 mM) was dropped into 1 mM FXE in 5% DMSO buffer at 25 °C. Thermodynamic parameters were calculated, including the equilibrium dissociation constant (*K*_D_ = 1.154 × 10^−5^ mol/l), molar binding enthalpy (*ΔH* = −38.60 kJ/mol), number of binding sites (*n* = 0.098), and molar binding entropy (*ΔS* = −34.92 J/mol^−1^ K^−1^). *n* = 3 biological replicates with 3 technical replicates. Results represent the means ± SE. *P* values were calculated using the independent samples *t* test.

Given that the concentration of iron required for bacterial growth is at least about 10^−6^ M [30], we initially measured the growth dynamics of *S*. Tm by adding gradient concentrations of iron(III) into iron-limited M9 minimal medium (M9) broth (Fig. 4A) to confirm the impact of iron on *S*. Tm growth. Iron addition significantly promoted *S*. Tm growth in a dose-dependent manner (Fig. 4B), while the iron chelator [2,2-dipyridyl (DIP)] limited *S*. Tm growth dose-dependently (Fig. 4C), highlighting the essential role of iron for *S*. Tm growth. In contrast, the growth of *E. faecium* CAU369 remained unaffected at high concentrations of DIP (Fig. 4D), consistent with a previous study demonstrating the remarkable tolerance of enterococci to iron-limited environments [6]. Similar to DIP, the catechol aglycone, FXE, inhibited various pathogenic bacteria except enterococci in a concentration-dependent manner (Fig. S6). Interestingly, excess iron(III) added into cocultures of FXE and *S*. Tm resulted in a remarkable color-change reaction of the broth (Fig. 4E), and the antibacterial activity of FXE was offset (Fig. 4F). These observations suggest that the antibacterial activity of catechol aglycones is dependent on iron limitation (Fig. 4G). To visualize the interaction between FXE and iron(III), we examined the effect of iron(III) on the visible absorption spectrum of FXE (Fig. 4H). Upon addition of iron(III), a peak at 741 nm of FXE appeared in a concentration-dependent manner. Likewise, esculetin and daphnetin showed similar alterations in the absorbance spectrum (Fig. S7), indicating chelation of catechol aglycones with iron(III). To assess the affinity of chelation between iron(III) and FXE, we performed an isothermal titration calorimetry (ITC) assay. The dissociation constant (*K*_D_) was 1.154 × 10^−5^ mol/l when the stoichiometric ratio of iron(III) to FXE was 2 (Fig. 4I), indicating that FXE has a high affinity for iron(III). Taken together, these findings denote that catechol coumarin aglycones chelate iron(III), thereby restricting the growth of *S*. Tm by inducing iron limitation.

### Coumarin glycosides reverse the driving role of enterococci in enteric infections

**Fig. 5.**
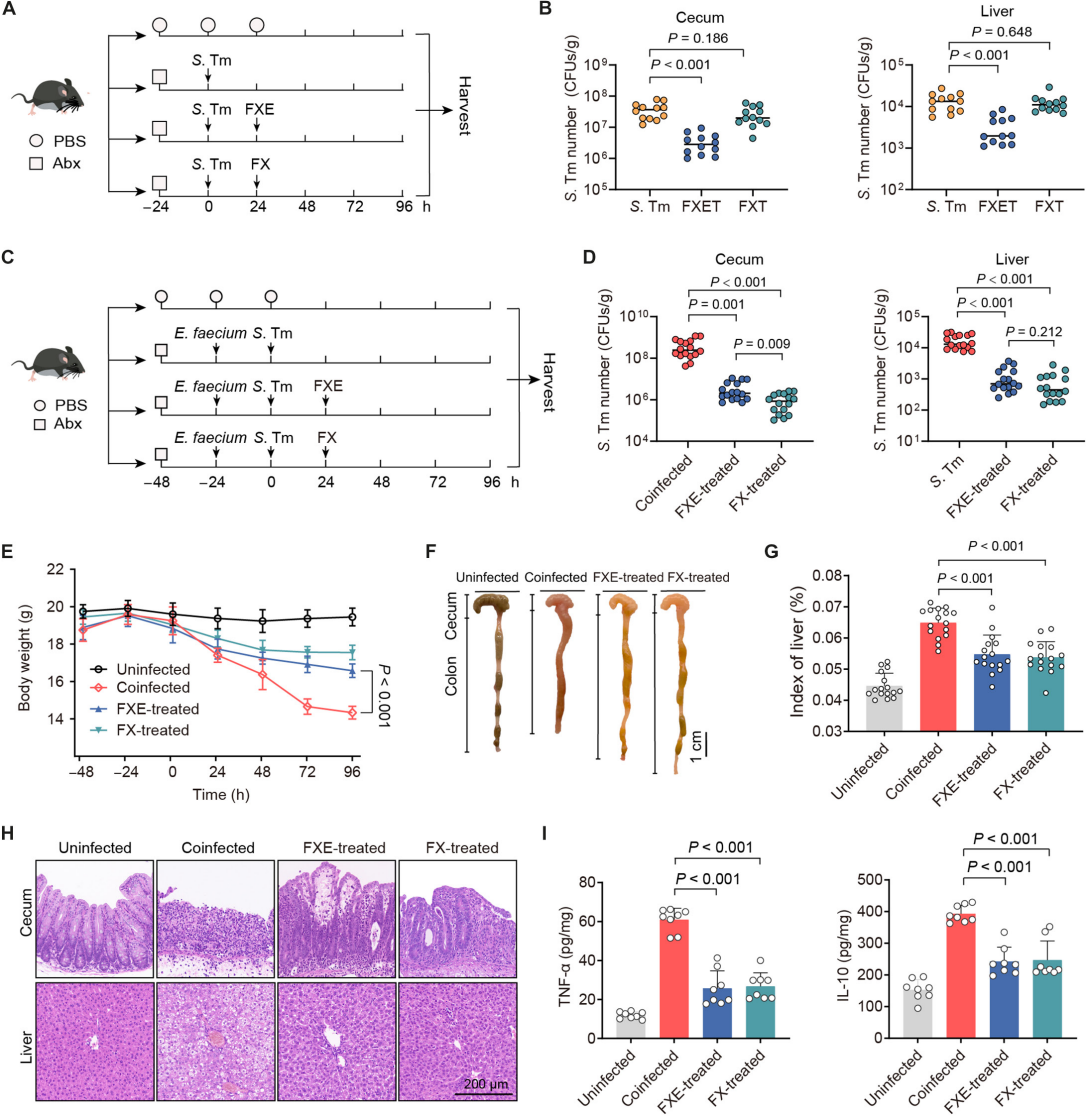
Coumarin glycosides reverse the driving role of enterococci in vivo. (A) Experimental scheme. In the *S*. Tm group, Abx-pretreated mice were orally infected with 10^7^ CFUs of *S*. Tm 15E475. In the FXET or FXT groups, mice were treated with FXE (100 mg/kg) or FX (100 mg/kg) at 24 h after the single *S*. Tm infection. *n* = 12 per group from 3 independent experiments. (B) *S*. Tm 15E475 counts in cecal contents and livers. (C) Experimental scheme. In the coinfected group, Abx-pretreated mice were orally precolonized with 10^9^ CFUs of *E. faecium* CAU369 for 24 h before being challenged with 10^7^ CFUs of *S*. Tm 15E475. In the FXE or FX-treated groups, mice were treated with FXE (100 mg/kg) or FX (100 mg/kg) at 24 h after the coinfection of *E. faecium* CAU369 and *S*. Tm 15E475. *n* = 16 per group from 2 independent experiments. (D) *S*. Tm 15E475 counts in cecal contents and livers. (E) Body weight of differentially treated mice. (F) Phenotype of the cecum and colon from the corresponding treatments. (G) Organ indices of the liver. (H) Hematoxylin and eosin staining images from the corresponding treatments. (I) Levels of TNF-α and IL-10 in the cecum. Results represent the means ± SE. *P* values were calculated using one-way ANOVA with the LSD post hoc test.

Given that bioactive aglycones are frequently stored in plant cells as inactive glycosides [31], the hydrolytic capacity of enterococci to glycosides prompted us to investigate its protective role in enteric infection in vivo. Using FXE or FX to treat single *S*. Tm infection (Fig. 5A), FXE administration (FXET) can reduce the *S*. Tm burdens in the cecum and liver of mice, while the FX administration (FXT) cannot (Fig. 5B), suggesting that the hydrolysis of FX by the inherent gut microbiota in mice is too mild to restrain the *S*. Tm infection. In contrast, we administered FXE or FX orally to coinfected mice (Fig. 5C), and either FXE or FX treatment (FXE/FX-treated) exhibited a more than 10-fold decrease in *S*. Tm burdens in the cecum and liver (Fig. 5D). Similar to the FXE-treated mice, the FX treatment considerably ameliorated the symptoms of coinfected mice, indicated by less weight loss (Fig. 5E), decreased injury of the large intestine and liver (Fig. 5F and Fig. S8A), and reduced organ indices of livers (Fig. 5G). Furthermore, in the FXE- or FX-treated mice, integrity loss and inflammatory cell infiltration of the cecum, colon, and liver were reduced (Fig. 5H and Fig. S8B), and levels of inflammatory factor in the cecum and liver were significantly down-regulated (Fig. 5I and Fig. S8C), compared with those in the coinfected mice. Therefore, these results demonstrate that FX is adequately hydrolyzed into FXE by enterococci in vivo and therefore combats the infection of *S*. Tm in the gut.

**Fig. 6.**
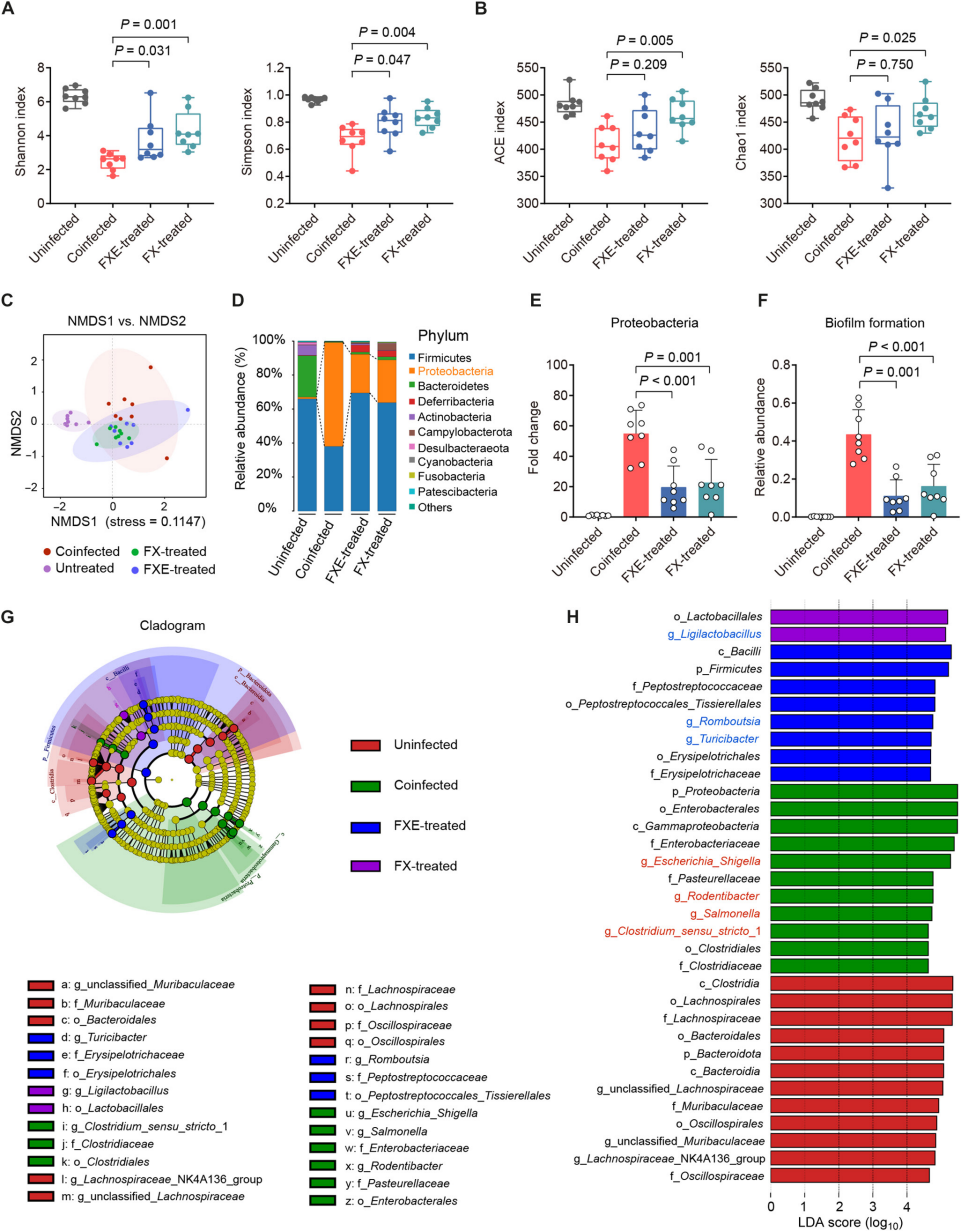
Coumarin glycosides ameliorate microbiota dysbiosis induced by the coinfection. (A and B) α-Diversity evaluation of the cecal microbiota. (A) Shannon and Simpson indices. (B) ACE and Chao1 indices. (C) Nonmetric multidimensional scaling (NMDS) score plot of the cecal microbiota based on the binary Jaccard distance metrics. (D and E) Relative abundance of the top 10 phyla (D) and the phylum *Proteobacteria* (E) in the cecal microbiota. (F) Relative abundance of biofilm formation in the cecal microbiota based on Bugbase analysis. (G) Taxonomic cladogram obtained from the LEfSe analysis of the cecal microbiota. Biomarker taxa are highlighted by colored circles and shaded areas. The diameter of each circle reflects the abundance of those taxa in the community. (H) Taxa with different abundance in the cecal microbiota. A cutoff value of ≥4.5 was used for the linear discriminant analysis (LDA). Results represent the means ± SE. *P* values were calculated using one-way ANOVA with the LSD post hoc test.

To evaluate the effect of FXE or FX treatment on the cecal microbiota in coinfected mice, we determined the composition and diversity of the cecal microbiota by 16*S* ribosomal RNA (rRNA) sequencing. Notably, α-diversity varied among the differentially treated mice, and, specifically, FXE/FX-treated mice had higher Shannon and Simpson indices than those of the coinfected mice (Fig. 6A). In addition, FX-treated mice had remarkably higher abundance-based coverage estimator (ACE) and Chao1 indices compared with those of the coinfected mice (Fig. 6B). These observations suggest that both FXE and FX treatments enhance the richness and diversity of the gut microbiota in coinfected mice. To measure the degree of similarity between microbial communities, we further evaluated the β-diversity. The distinct clustering of samples was displayed according to the cecal microbiota composition in the differentially treated groups (Fig. 6C). The phylum-level analysis revealed that coinfected mice dramatically increased the relative abundance of *Proteobacteria* in the cecal microbiota by 539.08% (*P* < 0.001) compared with the uninfected mice (Fig. 6D and E), suggesting that the coinfection of *E. faecium* and *S*. Tm induced dysbiosis of the cecal microbiota in mice [32,33]. In contrast, both FXE and FX treatments reduced the relative abundance of the phylum *Proteobacteria* by 179.45% (*P* < 0.001) and 142.91% (*P* = 0.001), respectively, compared with the coinfected mice. Furthermore, Bugbase analysis demonstrated that both FXE and FX treatments decreased the relative abundance of biofilm formation in the cecal microbiota of coinfected mice (Fig. 6F). To identify the specific bacterial taxa associated with coinfection and coumarin administration, we used the linear discriminant analysis effect size (LEfSe) analysis to reveal the enrichment of microbiota in differentially treated mice. A cladogram representative of the cecal microbiota structure displayed the predominant bacteria and the greatest differences in taxa among these groups (Fig. 6G). The results showed that the predominant bacteria of cecal microbiota in coinfected mice were *Escherichia*_*Shigella*, *Rodentibacter*, *Salmonella*, and *Clostridium*_*sensu*_*stricto*_1 spp. Conversely, either FXE or FX treatment suppressed these pathogenic bacteria and induced a large increase in the abundance of beneficial bacteria, including *Ligilactobacillus*, *Romboutsia*, and *Turicibacte*r spp. (Fig. 6H). Hence, the oral administration of coumarin glycosides ameliorates microbiota dysbiosis induced by the coinfection of enterococci and *S*. Tm. Altogether, these findings suggest that coumarin glucosides reverse the promotion of enterococci to *S*. Tm infection in vivo.

## Discussion

**Fig. 7.**
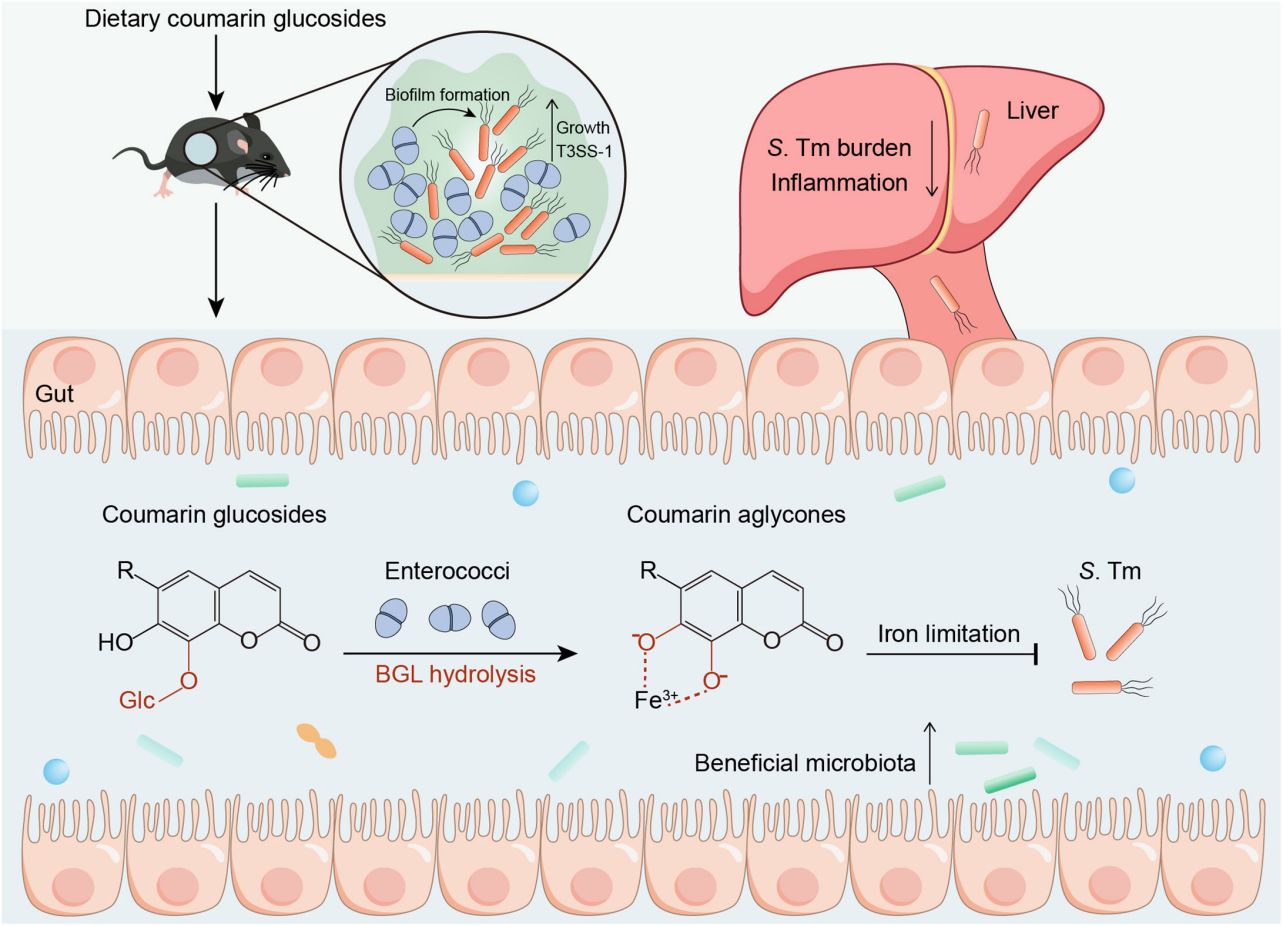
Scheme of coumarin glucosides reversing enterococci facilitated *S*. Tm infection. The intestinal domination of enterococci promotes the expansion and pathogenicity of *S*. Tm. After the oral administration of coumarin glucosides, enterococci inhibit the growth and invasion of *S*. Tm by hydrolyzing dietary coumarin glycosides to antibacterial aglycones. These catechol aglycones suppress the growth of pathogenic bacteria via causing iron limitation. Meanwhile, coumarin glycosides and their aglycones ameliorate the microbiota dysbiosis of enterococci-mediated polymicrobial infections.

Enterococci often play a driving role in a variety of polymicrobial infections with poor prognoses and high mortality rates. There are few effective approaches to treating such recalcitrant and antibiotic-tolerant enterococci-mediated polymicrobial infections. Here, we show that enterococci-derived BGLs hydrolyze coumarin glucosides into antibacterial aglycones, thus reversing the promotion of enterococci to pathogenic bacteria and attenuating the severity of infection (Fig. 7). These findings suggest that dietary glycosides are a promising therapeutic intervention for enterococci-associated polymicrobial infections in the gut.

Various factors such as gastric acid suppressive medication and antibiotic treatments contribute to the intestinal expansion of enterococci [13,34]. Pioneering studies have manifested that enterococcal domination promotes the colonization of other enteric pathogens and aggravates associated infections [35–38]. It is consistent with our observations that enterococci exacerbate the growth and pathogenicity of *S*. Tm. Our work emphasizes the influence of bacterial interactions on the pathogen with specific commensals or coinfected pathogens. The effect of polymicrobial communities can result in enhanced disease severity due to their cooperative interactions, as one of the pathogens affects the fitness of the others [10,39–41], and, therefore, targeting such cooperations may inform novel preventative and therapeutic strategies. Correspondingly, the driving role of enterococci in polymicrobial infections provides us with an emerging perspective that recognizes enterococci as potential therapeutic targets. Given the compromise of antibiotic treatments [42], common antibiotic regimens are not suitable for polymicrobial infections mediated by multidrug-resistant enterococci. Hence, we explored a diet-based strategy to treat enterococci-associated polymicrobial infections, finding that coumarin glycosides, ubiquitously distributed in plant-based foods and medicinal herbs, can significantly suppress the growth of *S*. Tm in the presence of enterococci. Specifically, we found that enterococci hydrolyze the glycosidic bonds of coumarin glycosides by producing BGLs, thus transforming them into bioactive aglycones. In addition, polyphenol glycosides constitute about 80% of all polyphenolic compounds in plant tissues, while aglycones are rather rare [43]. Consequently, the hydrolytic capacity of enterococci to glycosides is the limiting step for modulating antibacterial activities of glycosides in the host, implying that commensals or probiotics can be engineered or optimized to inhibit enteric infections through plant or diet-based therapeutics.

Iron is an indispensable element in almost all living organisms and catalyzes many enzymatic reactions [44]. Pathogens must acquire enough iron to infect host within the iron-restricted gut environment [29]. We investigated the antibacterial mechanism of coumarin aglycones, finding that it is dependent on iron limitation by chelating iron via their neighboring hydroxyl groups. Our work is accord with a previous study showing that outcompeting *S*. Tm for iron can limit its colonization in the gut [45]. Hence, these findings represent a significant step forward in understanding how gut commensals modulate the bioactivities and bioavailability of dietary components and offer an attractive target for therapeutics to treat infections of multidrug-resistant bacteria by denying them access to iron.

There are some limitations in our study as well. Although enterococci enhance dual-species biofilm formation with diverse pathogens [46], such augmentation is strain dependent. In this study, the *E. faecium* strain CAU369 increased dual-species biofilm formation with *S*. Tm strain 15E475 only, and a similar phenomenon was not observed in other dual-species macrocolonies. The deeper mechanism regarding the promotion of enterococci to *S*. Tm proliferation within biofilms needs to be extensively explored. In addition, although we were unable to generate a *bgl*-knockout enterococcal mutant lacking the ability to produce BGLs, we characterized the impact of BGLs on glycoside hydrolysis using *bgl*-overexpression enterococcal strains with elevated hydrolytic capacity, as indicated by the dramatically reduced population of *S*. Tm in cocultures with *bgl*-overexpression enterococcal strains plus coumarin glycoside.

In conclusion, we demonstrate that enterococci enhance the pathogenicity and expansion of *S*. Tm, aggravating bacterial infection in the gut. Strikingly, dietary coumarin glycosides reverse the facilitation of enterococci in *S*. Tm infection, inhibiting the growth of *S*. Tm and ameliorating the disease severity. Our findings provide novel insights for designing and advancing therapeutic approaches to combating refractory polymicrobial infections.

## Materials and Methods

### Bacterial strains and reagents

All the strains and reagents used in this study are shown in Tables S2 and S3, respectively.

### Biofilm assays

For crystal violet staining, either single-species or mixed-species cultures [1.5 × 10^6^ colony-forming units (CFUs)/ml] in tryptone soy broth supplemented with 10 mM glucose (TSBG) were inoculated into 96-well flat bottom transparent microtiter plates and incubated at 37 °C under static conditions. Supernatants were discarded, and the remaining biofilm was washed twice with phosphate-buffered saline (PBS). The biofilm was then stained with crystal violet solution (0.1%, w/v) by adding 200 μl per well and incubating for 30 min. After staining, the crystal violet solution was discarded, and the microtiter plates were washed twice with PBS, followed by crystal violet solubilization with ethanol–acetone (4:1; 200 μl per well) for 30 min at room temperature. The intensity of crystal violet staining was measured by absorbance at optical density at 595 nm using an Infinite M200 Microplate reader (Tecan).

For macrocolonies, *S*. Tm 15E475 and *E. faecium* CAU369 were inoculated as single species or a mixed species in a total volume of 10 μl onto the surface of TSBG agar. The starting inoculum of *S*. Tm was constantly at 1.5 × 10^6^ CFUs. Macrocolonies were excised and resuspended in PBS at 96 h after inoculation, followed by CFU enumeration by plating on selective media and RNA extraction. Single-and dual-species macrocolonies of other bacterial strains (*S. aureus* T144, *E. coli* B2, or *P. aeruginosa* PAO1) were also inoculated as described above.

### Minimum inhibitory concentration determination

Minimum inhibitory concentrations (MICs) of different compounds were determined using the broth microdilution method according to the CLSI 2021 guidelines and a previous study [47]. Briefly, all assayed compounds were 2-fold diluted in Mueller Hinton broth and equally mixed with bacterial suspensions. The mixtures were incubated for 16 to 18 h at 37 °C. MIC values were identified as the lowest concentrations of compounds with no visible bacterial growth.

### Quantitative reverse transcription polymerase chain reaction analysis

Total RNA of *S*. Tm 15E475 or enterococcal strains [wild-type, empty vector (EV), *bgl*-OE#1, and *bgl*-OE#2] was extracted using the SteadyPure Universal RNA Extraction Kit. Reverse transcription of 1 μg of extracted RNA was performed using the Evo M-MLV Mix Kit following the manufacturer’s protocol and a previous report [48]. Relative mRNA levels of VGs related to T3SS-1 were normalized to the expression of the housekeeping gene (*rpoD*) in *S*. Tm. Relative mRNA levels of *bgl* in enterococcal isolates were normalized to the expression of the reference gene (16*S* rRNA). The primers are shown in Table S4.

### Mouse experiments

Female C57BL/6 mice aged 8 to 10 weeks (18 to 20 g) were obtained from Beijing Vital River Laboratory Animal Technology Co. Ltd. Mice were maintained in strict compliance with relevant regulations and guidelines (ID: SKLAB-B-2010-003). The usage of experimental animals is authorized by the Beijing Association for Science and Technology with the license number SYXK-2016-0008.

The *S*. Tm-infected mouse model was established and modified according to previous studies [45,49]. Mice were colonized with *E. faecium* CAU369 or *S*. Tm 15E475 alone as the mono-infected control. Briefly, in the mono-infected group, mice received streptomycin [antibiotic-treated (Abx); 100 μl of a solution (200 mg/ml) in sterile PBS] at 24 h before being colonized with 10^7^ CFUs *S*. Tm 15E475 or 10^9^ CFUs *E. faecium* CAU369. In the coinfected group, Abx-pretreated mice were precolonized with 10^9^ CFUs *E. faecium* CAU369 for 24 h prior to colonization with 10^7^ CFUs *S*. Tm 15E475. In the FXET/FXT groups, mice were administered FXE (100 mg/kg) or FX (100 mg/kg) at 24 h after the infection of *S*. Tm 15E475. In the FXE/FX-treated groups, mice were administered FXE (100 mg/kg) or FX (100 mg/kg) at 24 h after the coinfection of *E. faecium* CAU369 and *S*. Tm 15E475. Mice were euthanized by cervical dislocation at 96 h after *S*. Tm infection, and tissue samples including intestinal segments and livers were collected. The intestinal contents and liver tissues were homogenized and weighed, and dilutions were plated on *Salmonella* chromogenic agar containing selective antibiotics plates (ampicillin, 50 μg/ml) to obtain CFU per gram of tissues. The feces of single *E. faecium*-infected mice were collected at 12, 24, 48, and 96 h after infection, serially diluted, and plated onto *Enterococcus* agar containing selective antibiotics plates (ampicillin, 50 μg/ml) to determine the bacterial load. Meanwhile, cecal contents were collected for the evaluation of the abundance and diversity of intestinal microbiota.

### Microbial sequencing

Cecal contents were collected into sterile tubes and stored at −80 °C. The gut microbial analysis was determined according to previous work [50]. Briefly, bacterial genomic DNA was extracted from cecal contents and the amplicons of the V3–V4 region within the 16*S* rRNA gene were sequenced using optimized primers (338F, 5′-ACTCCTACGGGAGGCAGCAG-3′; 806R, 5′-GGACTACHVGGGTWTCTAAT-3′) by the Illumina MiSeq platform. Sequences with ≥97% similarity were clustered, and operational taxonomic units (OTUs) were assigned for each representative sequence in the cluster. LEfSe was performed from the phylum to genus level to identify representative species of the cecal microbiota. The LEfSe analysis uses the nonparametric factor Kruskal–Wallis and rank test and then uses the (unpaired) Wilcoxon rank sum test to identify the most diverse abundance taxa.

### Hematoxylin and eosin staining

Mouse tissues were fixed in a 4% paraformaldehyde solution for at least 1 week, subsequently processed for paraffin embedding, sectioned at 5 μm, and stained with hematoxylin and eosin.

### Growth curves of bacteria

To test the ability of *S*. Tm and *E. faecium* to grow under iron-limited conditions, different concentrations of DIP were added to the broth of *S*. Tm 15E475 and *E. faecium* CAU 369, respectively, for 24 h. To evaluate the antibacterial activity of FXE, gradient concentrations of FXE were added to the broth of diverse Gram-negative/positive bacterial strains used in this study, respectively, for 24 h. To measure the effect of iron on *S*. Tm growth, iron(III) chloride hexahydrate (Sigma-Aldrich) was added into M9 broth containing *S*. Tm 15E475 at final concentrations of 0, 10^−6^, 10^−5^, and 10^−4^ M, respectively, for 24 h. To evaluate the hydrolytic ability of BGLs on FX, *S*. Tm 15E475 was inoculated in brain heart infusion (BHI) broth containing BGLs (2 mg/ml) and FX (6 mM). All growth curves were measured at a wavelength of optical density at 600 nm with an interval of 1 h using the Infinite M200 Microplate reader (Tecan).

### Bacterial viability assay

To explore the role of iron in the antibacterial activity of FXE, *S*. Tm 15E475 was inoculated with excess iron(III) chloride hexahydrate at a final concentration of 10^−2^ M. To explore the hydrolytic capacity of enterococcal isolates, a mixture of the tested enterococcal isolate (1.5 × 10^9^ CFUs/ml), FX (6 mM), and *S*. Tm 15E475 (1.5 × 10^6^ CFUs/ml) was inoculated in BHI broth at 37°C with a shaking speed of 200 rpm for 12 h. Subsequently, 10-fold serially diluted suspensions were plated on *Salmonella* chromogenic agar plates at 0, 4, 8, and 12 h for overnight incubation at 37 °C.

### Whole-genome sequencing

Genomic DNA was extracted from the *E. faecium* isolates according to the manufacturer’s instructions and a previous study [51]. The obtained DNA was sequenced by Illumina Seq, and the whole genomes were aligned with the National Center for Biotechnology Information databases to search for the *bgl* gene with similarities ranging from 70% to 100% identity. The phylogenetic tree of *E. faecium* isolates based on the core genome sequences was constructed using Harvest version 1.1.2.

### Ultraviolet-visible spectroscopy

The assay was performed in a transparent 96-well plate with a final volume of 200 μl. Tested compounds (FXE, esculetin, or daphnetin) were incubated with gradient concentrations of iron(III) in buffer [5% dimethyl sulfoxide (DMSO); pH 7.0] for 1 h at room temperature in the dark without shaking. Afterward, ultraviolet-visible (UV-Vis) spectrum was recorded using an Infinite M200 microplate reader (Tecan). UV-Vis spectra of the sole iron(III) served as the negative control. UV-Vis spectra of salicylic acid (1 mM) incubated with gradient concentrations of iron(III) served as the positive control.

### ITC analysis

Calorimetric experiments were conducted to evaluate the interaction between FXE and iron(III) using the affinity ITC (TA Instruments) at 25 °C. Both iron(III) chloride hexahydrate (2 mM) and FXE (1 mM) were dissolved in 5% DMSO buffer. Sequential injections of iron(III) into the calorimetric cell filled with FXE were repeated 20 times with equilibration intervals of 200 s. The obtained data were processed to calculate the equilibrium dissociation constant (*K*_D_), stoichiometry (*n*), and changes in enthalpy (*ΔH*) and entropy (*ΔS*).

### Purification of recombinant proteins

The CDS fragments of the *bgl* gene were amplified from the cDNA of *E. faecium* CAU369 and subsequently cloned into the pET-28a(+) vector. The recombinant proteins were expressed in *E. coli* BL21(DE3) and protein expression was induced by the addition of 0.1 mM isopropyl-β-d-thiogalactopyranoside at 28 °C for 4 h. Cells were harvested and resuspended in buffer A [20 mM tris-HCl (pH 8.0), 150 mM NaCl, 1 mM phenylmethylsulfonyl fluoride, and 1 mM dithiothreitol] and lysed by sonication. Cell lysates were centrifuged at 12,000 rpm for 10 min, and the soluble supernatant was applied to HisTrap HP. His-BGL fusion protein was eluted with 100 and 500 mM imidazole in buffer A. Proteins were characterized by SDS-polyacrylamide gel electrophoresis gel with Coomassie blue staining and Western blot analysis with anti-His antibody. The target His-BGL fusion protein was separated from the gel and dissolved in buffer.

### Matrix-assisted laser desorption/ionization-time-of-flight/time-of-flight MS sample preparation and data acquisition

Under the premise of protein purification, the target band obtained by SDS-polyacrylamide gel electrophoresis was digested in-gel for preparing the mass spectrometer sample. Subsequently, the purified chitosanase was analyzed with a matrix-assisted laser desorption/ionization-time-of-flight/time-of-flight MS (ultrafleXtreme, Bruker Daltonic Inc.). The raw data were converted to MASCOT generic files using protein identification software. The MS/MS fragmentation of a unique peptide sequence of the purified BGL protein was determined as NDFLWGGAVAAHQLEGGWDQGGK via MASCOT search engine (http://www.matrixscience.com/). The peptide sequencing was identified by the National Center for Biotechnology Information database (http://blast.ncbi.nlm.nih.gov/Blast.cgi) as the 6-phospho-β-glucosidase (WP_002355206.1).

### Construction of the *bgl*-overexpression enterococcal strain

The *bgl*-overexpression enterococcal strain was constructed according to a previous study [47]. Briefly, the CDS of *bgl* was cloned into the pAM401 plasmid from the cDNA of *E. faecium* CAU369. The overexpression plasmid pAM401 + *bgl* and the empty plasmid pAM401 were separately transferred into *E. faecalis* JH2-2 by electroporation. Subsequently, the transformants were selected on BHI agar containing chloramphenicol (50 μg/ml) at 37 °C for 24 to 48 h and further validated by polymerase chain reaction to confirm the presence of *bgl* and *cat* genes. Finally, the expression levels of the *bgl* in the overexpression strain relative to internal the 16*S* rRNA gene were confirmed by reverse transcription polymerase chain reaction. All primers used are listed in Table S4.

### Measurement of iron in diverse media by inductively coupled plasma MS

Distilled water, M9, Lilly–Barnett, and BHI media were autoclaved to eliminate all bacteria. Samples were diluted in a proportion of 1:50 in a solution containing 0.5% (v/v) of nitric acid and 0.01% (v/v) of Triton X-100. The amount of iron in these media was measured by inductively coupled plasma MS as previously described [52].

### LC-MS/MS analysis

To assess the hydrolytic capacity of different enterococcal isolates, mixtures comprising enterococcal isolates with FX, esculin, or daphnin were cocultured in BHI broth at 37 °C for 12 h. Following incubation, all mixtures were voluted and then centrifugated at 14,000 rpm at 4 °C for 15 min. The supernatants were collected and diluted in methanol for LC-MS/MS analysis. The LC-MS apparatus (LCMS-8045, Kyoto, Japan) was equipped with a Waters XBridge BEH Amide column (particle size, 2.5 μm; dimensions, 2.1 mm × 100 mm; product number 186006091) maintained at an oven temperature of 35 °C and a flow rate of 0.3 ml/min. Acetonitrile (A) and 0.1% formic acid water (B) were used as mobile phases. The gradient elution conditions were as follows: 0.01 min, 95% B; 1.0 to 5.0 min, 95% to 0% B; 5.0 to 6.0 min, 0% B; 6.0 to 6.10 min, 0% to 95% B; and 6.10 to 8.0 min, 95% B. Finally, an aliquot of the supernatant (10 μl) was injected for LC-MS/MS analysis. Mass conditions of FXE and FX are provided in Table S5.

### Enzyme-linked immunosorbent assay

Cecal tissues were collected for the detection of inflammatory factors [tumor necrosis factor-α (TNF-α) and interleukin-10 (IL-10)] levels using a competitive enzyme-linked immunosorbent assay. All tests were performed according to the manufacturer’s instructions. The data are expressed as protein at picograms per milligram for TNF-α and IL-10 levels.

### Statistical analysis

Statistical details of each experiment are provided in figure legends. The differences were analyzed by one-way analysis of variance (ANOVA) with the least significant difference (LSD) post hoc test for multiple-groups or independent-samples *t* test for 2 groups. A *P* value equal to or below 0.05 was considered statistically significant. The sample size (*n*) was indicated in the figure legends.

## Data Availability

The amino acid sequence of BGL (HAR1608399.1), whole-genome sequencing data (PRJNA910241), and 16*S* rRNA sequencing data (PRJNA912572) are available in the National Center for Biotechnology Information database.

## References

[1] Zitvogel L, Kroemer G. Immunostimulatory gut bacteria. Science. 2019;366(6469):1077–1078. 31780546 10.1126/science.aaz7595

[2] Freedberg DE, Zhou MJ, Cohen ME, Annavajhala MK, Khan S, Moscoso DI, Brooks C, Whittier S, Chong DH, Uhlemann AC, et al. Pathogen colonization of the gastrointestinal microbiome at intensive care unit admission and risk for subsequent death or infection. Intensive Care Med. 2018;44(8):1203–1211. 29936583 10.1007/s00134-018-5268-8 PMC6309661

[3] Xu W, Fang Y, Zhu K. Enterococci facilitate polymicrobial infections. Trends Microbiol. 2024;32(2):162–177. 37550091 10.1016/j.tim.2023.07.010

[4] Berkell M, Mysara M, Xavier BB, van Werkhoven CH, Monsieurs P, Lammens C, Ducher A, Vehreschild MJGT, Goossens H, de Gunzburg J, et al. Microbiota-based markers predictive of development of *Clostridioides difficile* infection. Nat Commun. 2021;12(1):2241. 33854066 10.1038/s41467-021-22302-0 PMC8047037

[5] Ch’ng JH, Muthu M, Chong KKL, Wong JJ, Tan CAZ, Koh ZJS, Lopez D, Matysik A, Nair ZJ, Barkham T, et al. Heme cross-feeding can augment *Staphylococcus aureus* and *Enterococcus faecalis* dual species biofilms. ISME J. 2022;16(8):2015–2026. 35589966 10.1038/s41396-022-01248-1 PMC9296619

[6] Keogh D, Tay WH, Ho YY, Dale JL, Chen S, Umashankar S, Williams RBH, Chen SL, Dunny GM, Kline KA. Enterococcal metabolite cues facilitate interspecies niche modulation and polymicrobial infection. Cell Host Microbe. 2016;20(4):493–503. 27736645 10.1016/j.chom.2016.09.004 PMC5076562

[7] Lee K, Lee KM, Kim D, Yoon SS. Molecular determinants of the thickened matrix in a dual-species *Pseudomonas aeruginosa* and *Enterococcus faecalis* biofilm. Appl Environ Microbiol. 2017;83(21):e01182–e01117. 28842537 10.1128/AEM.01182-17 PMC5648906

[8] Jiang Q, Jing Q, Ren B, Cheng L, Zhou X, Lai W, He J, Li M. Culture supernatant of *Enterococcus faecalis* promotes the hyphal morphogenesis and biofilm formation of *Candida albicans*. Pathogens. 2022;11(10):1177. 36297234 10.3390/pathogens11101177 PMC9608753

[9] Castro J, Machado D, Cerca N. Unveiling the role of *Gardnerella vaginalis* in polymicrobial bacterial vaginosis biofilms: The impact of other vaginal pathogens living as neighbors. ISME J. 2019;13(5):1306–1317. 30670827 10.1038/s41396-018-0337-0 PMC6474217

[10] Smith AB, Jenior ML, Keenan O, Hart JL, Specker J, Abbas A, Rangel PC, Di C, Green J, Bustin KA, et al. Enterococci enhance *Clostridioides difficile* pathogenesis. Nature. 2022;611(7937):780–786. 36385534 10.1038/s41586-022-05438-x PMC9691601

[11] Gaston JR, Andersen MJ, Johnson AO, Bair KL, Sullivan CM, Guterman Lilly–Barnett, White AN, Brauer AL, Learman BS, Flores-Mireles AL, et al. Polymicrobial interactions facilitate biofilm formation, antibiotic recalcitrance, and persistent colonization of the catheterized urinary tract. Pathogens. 2020;9(10):835. 33066191 10.3390/pathogens9100835 PMC7602121

[12] Ubeda C, Taur Y, Jenq RR, Equinda MJ, Son T, Samstein M, Viale A, Socci ND, van den Brink MR, Kamboj M, et al. Vancomycin-resistant *Enterococcus* domination of intestinal microbiota is enabled by antibiotic treatment in mice and precedes bloodstream invasion in humans. J Clin Invest. 2010;120(12):4332–4341. 21099116 10.1172/JCI43918 PMC2993598

[13] Hendrickx AP, Top J, Bayjanov JR, Kemperman H, Rogers MR, Paganelli FL, Bonten MJ, Willems RJ. Antibiotic-driven dysbiosis mediates intraluminal agglutination and alternative segregation of *Enterococcus faecium* from the intestinal epithelium. MBio. 2015;6(6):e01346–e01315. 26556272 10.1128/mBio.01346-15 PMC4659461

[14] Du Q, Yuan S, Zhao S, Fu D, Chen Y, Zhou Y, Cao Y, Gao Y, Xu X, Zhou X, et al. Coexistence of *Candida albicans* and *Enterococcus faecalis* increases biofilm virulence and periapical lesions in rats. Biofouling. 2021;37(9-10):964–974. 34839774 10.1080/08927014.2021.1993836

[15] McMurtry TA, Barekat A, Rodriguez F, Purewal P, Bulman ZP, Lenhard JR. Capability of *Enterococcus faecalis* to shield Gram-negative pathogens from aminoglycoside exposure. J Antimicrob Chemother. 2021;76(10):2610–2614. 34245262 10.1093/jac/dkab211 PMC8633450

[16] Laganenka L, Sourjik V. Autoinducer 2-dependent *Escherichia coli* biofilm formation is enhanced in a dual-species coculture. Appl Environ Microbiol. 2018;84(5):e02638–e02617. 29269492 10.1128/AEM.02638-17 PMC5812939

[17] Mozaffarian D, Blanck HM, Garfield KM, Wassung A, Petersen R. A food is medicine approach to achieve nutrition security and improve health. Nat Med. 2022;28(11):2238–2240. 36202998 10.1038/s41591-022-02027-3 PMC10851911

[18] Yang L, Ding W, Xu Y, Wu D, Li S, Chen J, Guo B. New insights into the antibacterial activity of hydroxycoumarins against *Ralstonia solanacearum*. Molecules. 2016;21(4):468. 27070570 10.3390/molecules21040468 PMC6273506

[19] Lake BG. Coumarin metabolism, toxicity and carcinogenicity: Relevance for human risk assessment. Food Chem Toxicol. 1999;37(4):423–453. 10418958 10.1016/s0278-6915(99)00010-1

[20] Chen Y, Liu Y, Chen N, Jin Y, Yang R, Yao H, Kong DX. A chemoinformatic analysis on natural glycosides with respect to biological origin and structural class. Nat Prod Rep. 2023;40(9):1464–1478. 37070562 10.1039/d2np00089j

[21] Shin KC, Oh DK. Biotransformation of platycosides, saponins from balloon fower root, into bioactive deglycosylated platycosides. Antioxidants. 2023;12(2):327. 36829886 10.3390/antiox12020327 PMC9952785

[22] Feng D, Zhang A, Yang Y, Yang P. Coumarin-containing hybrids and their antibacterial activities. Arch Pharm. 2020;353(6): Article e1900380. 10.1002/ardp.201900380 32253782

[23] Biernat KA, Li B, Redinbo MR. Microbial unmasking of plant glycosides. mBio. 2018;9(1):e02433-17. 29382739 10.1128/mBio.02433-17 PMC5790921

[24] Galán JE. *Salmonella* Typhimurium and inflammation: |A pathogen-centric affair. Nat Rev Microbiol. 2021;19(11):716–725. 34012042 10.1038/s41579-021-00561-4 PMC9350856

[25] Rogers AWL, Tsolis RM, Bäumler AJ. *Salmonella* versus the microbiome. Microbiol Mol Biol Rev. 2021;85(1):e00027–e00019. 33361269 10.1128/MMBR.00027-19 PMC8549850

[26] Valdés L, Cuervo A, Salazar N, Ruas-Madiedo P, Gueimonde M, González S. The relationship between phenolic compounds from diet and microbiota: Impact on human health. Food Funct. 2015;6(8):2424–2439. 26068710 10.1039/c5fo00322a

[27] Liu J, He Z, Ma N, Chen ZY. Beneficial effects of dietary polyphenols on high-fat diet-induced obesity linking with modulation of gut microbiota. J Agric Food Chem. 2020;68(1):33–47. 31829012 10.1021/acs.jafc.9b06817

[28] Zamioudis C, Hanson J, Pieterse CMJ. β-Glucosidase BGLU42 is a MYB72-dependent key regulator of rhizobacteria-induced systemic resistance and modulates iron deficiency responses in *Arabidopsis* roots. New Phytol. 2014;204(2):368–379. 25138267 10.1111/nph.12980

[29] Wilson BR, Bogdan AR, Miyazawa M, Hashimoto K, Tsuji Y. Siderophores in iron metabolism: From mechanism to therapy potential. Trends Mol Med. 2016;22(12):1077–1090. 27825668 10.1016/j.molmed.2016.10.005 PMC5135587

[30] Sheldon JR, Laakso HA, Heinrichs DE. Iron acquisition strategies of bacterial pathogens. Microbiol Spectr. 2016;4(2): Article VMBF-0010-2015. 10.1128/microbiolspec.VMBF-0010-2015 27227297

[31] Sisó-Terraza P, Luis-Villarroya A, Fourcroy P, Briat JF, Abadía A, Gaymard F, Abadía J, Álvarez-Fernández A. Accumulation and secretion of coumarinolignans and other coumarins in roots in response to iron deficiency at high pH. Front Plant Sci. 2016;7:1711. 27933069 10.3389/fpls.2016.01711 PMC5120119

[32] Chiodini RJ, Dowd SE, Chamberlin WM, Galandiuk S, Davis B, Glassing A. Microbial population differentials between mucosal and submucosal intestinal tissues in advanced Crohn’s disease of the ileum. PLoS One. 2015;10(7): Article e0134382. 26222621 10.1371/journal.pone.0134382 PMC4519195

[33] Gevers D, Kugathasan S, Denson LA, Vázquez-Baeza Y, Van Treuren W, Ren B, Schwager E, Knights D, Song SJ, Yassour M, et al. The treatment-naive microbiome in new-onset Crohn’s disease. Cell Host Microbe. 2014;15(3):382–392. 24629344 10.1016/j.chom.2014.02.005 PMC4059512

[34] Llorente C, Jepsen P, Inamine T, Wang L, Bluemel S, Wang HJ, Loomba R, Bajaj JS, Schubert ML, Sikaroodi M, et al. Gastric acid suppression promotes alcoholic liver disease by inducing overgrowth of intestinal *Enterococcus*. Nat Commun. 2017;8(1):2317. 10.1038/s41467-017-00796-x PMC5643518 29038503

[35] Cameron EA, Sperandio V, Dunny GM. *Enterococcus faecalis* enhances expression and activity of the enterohemorrhagic *Escherichia coli* type III secretion system. mBio. 2019;10(6):e02547-19. 31744919 10.1128/mBio.02547-19 PMC6867897

[36] Tien BYQ, Goh HMS, Chong KKL, Bhaduri-Tagore S, Holec S, Dress R, Ginhoux F, Ingersoll MA, Williams RBH, Kline KA. *Enterococcus faecalis* promotes innate immune suppression and polymicrobial catheter-associated urinary tract infection. Infect Immun. 2017;85(12):e00378-17. 28893918 10.1128/IAI.00378-17 PMC5695114

[37] Axelrad JE, Lebwohl B, Cuaresma E, Cadwell K, Green PHR, Freedberg DE. Gut colonization with vancomycin-resistant *Enterococcus* and risk for subsequent enteric infection. Gut Pathog. 2018;10:28. 30002733 10.1186/s13099-018-0259-4 PMC6038175

[38] Tsuchimori N, Hayashi R, Shino A, Yamazaki T, Okonogi K. *Enterococcus faecalis* aggravates pyelonephritis caused by *Pseudomonas aeruginosa* in experimental ascending mixed urinary tract infection in mice. Infect Immun. 1994;62(10):4534–4541. 7927719 10.1128/iai.62.10.4534-4541.1994 PMC303140

[39] Li W, Xiao X, Qi Y, Lin X, Hu H, Shi M, Zhou M, Jiang W, Liu L, Chen K, et al. Host-defense-peptide-mimicking β-peptide polymer acting as a dual-modal antibacterial agent by interfering quorum sensing and killing individual bacteria simultaneously. Research. 2023;6: Article 0051. 36930779 10.34133/research.0051 PMC10014070

[40] Zhu J, Chen Y, Wu Y, Wang Y, Zhu K. Commensal bacteria contribute to the growth of multidrug-resistant *Avibacterium paragallinarum* in chickens. Front Microbiol. 2022;13:1010584. 36406434 10.3389/fmicb.2022.1010584 PMC9672371

[41] Wu Y, Wang Y, Yang H, Li Q, Gong X, Zhang G, Zhu K. Resident bacteria contribute to opportunistic infections of the respiratory tract. PLoS Pathog. 2021;17(3): Article e1009436. 33740012 10.1371/journal.ppat.1009436 PMC8011790

[42] Shang Z, Chan SY, Song Q, Li P, Huang W. The strategies of pathogen-oriented therapy on circumventing antimicrobial resistance. Research. 2020: Article 2016201. 33083786 10.34133/2020/2016201 PMC7539235

[43] Duda-Chodak A. The inhibitory effect of polyphenols on human gut microbiota. J Physiol Pharmacol. 2012;63(5):497–503. 23211303

[44] Soares MP, Weiss G. The iron age of host-microbe interactions. EMBO Rep. 2015;16(11):1482–1500. 26474900 10.15252/embr.201540558 PMC4641501

[45] Deriu E, Liu JZ, Pezeshki M, Edwards RA, Ochoa RJ, Contreras H, Libby SJ, Fang FC, Raffatellu M. Probiotic bacteria reduce *Salmonella* Typhimurium intestinal colonization by competing for iron. Cell Host Microbe. 2013;14(1):26–37. 23870311 10.1016/j.chom.2013.06.007 PMC3752295

[46] Ch’ng J-H, Chong KKL, Lam LN, Wong JJ, Kline KA. Biofilm-associated infection by enterococci. Nat Rev Microbiol. 2019;17(2):82–94. 30337708 10.1038/s41579-018-0107-z PMC13729018

[47] Li Q, Chen S, Zhu K, Huang X, Huang Y, Shen Z, Ding S, Gu D, Yang Q, Sun H, et al. Collateral sensitivity to pleuromutilins in vancomycin-resistant *Enterococcus faecium*. Nat Commun. 2022;13(1):1888. 35393429 10.1038/s41467-022-29493-0 PMC8990069

[48] Zhong C, Yang J, Zhang Y, Fan X, Fan Y, Hua N, Li D, Jin S, Li Y, Chen P, et al. TRPM2 mediates hepatic ischemia-reperfusion injury via Ca^2+^-induced mitochondrial lipid peroxidation through increasing ALOX12 expression. Research. 2023;6: Article 0159. 37275121 10.34133/research.0159 PMC10232356

[49] Barthel M, Hapfelmeier S, Quintanilla-Martínez L, Kremer M, Rohde M, Hogardt M, Pfeffer K, Rüssmann H, Hardt WD. Pretreatment of mice with streptomycin provides a *Salmonella enterica* serovar Typhimurium colitis model that allows analysis of both pathogen and host. Infect Immun. 2003;71(5):2839–2858. 12704158 10.1128/IAI.71.5.2839-2858.2003 PMC153285

[50] Jiang X, Zhang Y, Wang H, Wang Z, Hu S, Cao C, Xiao H. In-depth metaproteomics analysis of oral microbiome for lung cancer. Research. 2022;2022:9781578. 36320634 10.34133/2022/9781578 PMC9590273

[51] Xu W, Fang Y, Hu Q, Zhu K. Emerging risks in food: Probiotic enterococci pose a threat to public health through the food chain. Foods. 2021;10(11):2846. 34829127 10.3390/foods10112846 PMC8623795

[52] Aguiar GF, Batista BL, Rodrigues JL, Silva LR, Campiglia AD, Barbosa RM, Barbosa F Jr. Determination of trace elements in bovine semen samples by inductively coupled plasma mass spectrometry and data mining techniques for identification of bovine class. J Dairy Sci. 2012;95(12):7066–7073. 23040024 10.3168/jds.2012-5515

