# Supplementary material for: Coumarin Glycosides Reverse Enterococci-Facilitated Enteric Infections
Source: Research (Wash D C). 2024 May 16;7:0374. doi: 10.34133/research.0374 (PMC11096794; doi:10.34133/research.0374)
Supplement: Supplementary 1 — Figs. S1 to S8 Tables S1 to S5 [file research.0374.f1.zip › Supplementary Materials.docx]

**Supplementary Materials**

**Coumarin Glycosides Reverse Enterococci-facilitated Enteric Infections**

Wenjiao Xu^1, 2^, GuixinYuan^3^, Yuwen Fang^1,2^, Xiaojia Liu^1,2^, Xiaowei Ma^1,2^, and Kui Zhu^1,2^*

^1^ National Key Laboratory of Veterinary Public Health and Safety, College of Veterinary Medicine, China Agricultural University, Beijing 100193, China

^2^ Engineering Research Center of Animal Innovative drugs and Safety Evaluation, Ministry of Education, College of Veterinary Medicine, China Agricultural University, Beijing 100193, China.

^3^ Ministry of Agriculture and Rural Affairs Key Laboratory for Crop Pest Monitoring and Green Control, China Agricultural University, Beijing, 100193, China.

*Corresponding author: Kui Zhu; zhuk@cau.edu.cn

**Table of Contents**

**Tables**

Table S1. ---------------------------------------------------------------------------------------------------- 3

Table S2. ---------------------------------------------------------------------------------------------------- 8

Table S3. ---------------------------------------------------------------------------------------------------- 9

Table S4. ---------------------------------------------------------------------------------------------------- 10

Table S5. ---------------------------------------------------------------------------------------------------- 11

**Figures**

Figure S1. ---------------------------------------------------------------------------------------------------12

Figure S2. ---------------------------------------------------------------------------------------------------13

Figure S3. ---------------------------------------------------------------------------------------------------14

Figure S4. ---------------------------------------------------------------------------------------------------15

Figure S5. ---------------------------------------------------------------------------------------------------16

Figure S6. ---------------------------------------------------------------------------------------------------17

Figure S7. ---------------------------------------------------------------------------------------------------18

Figure S8. ---------------------------------------------------------------------------------------------------19

**Reference**------------------------------------------------------------------------------------------------------20

**Table S1. Antibacterial activities of coumarins and their analogues.**

| Chemical  structure | Analogues | *S*. Tm  15E475 | *E. coli* ATCC 25922 | | *S. aureus*  ATCC 29213 | | *E. faecium*  CAU369 | |
| --- | --- | --- | --- | --- | --- | --- | --- | --- |
|  |  | MIC (mg/mL) | | | | | | |
| 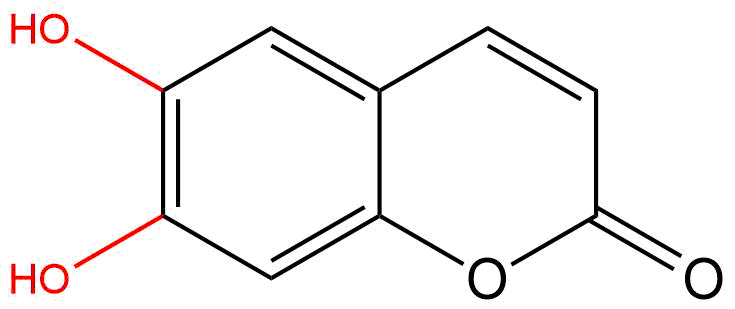 | Esculetin | **1.28** | | **0.64** | | **1.28** | | >5.12 |
| 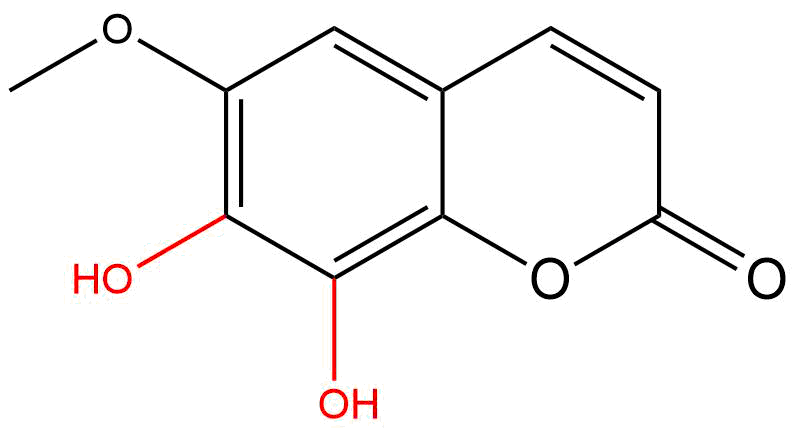 | Fraxetin | **0.64** | | **0.32** | | **0.64** | | >5.12 |
| 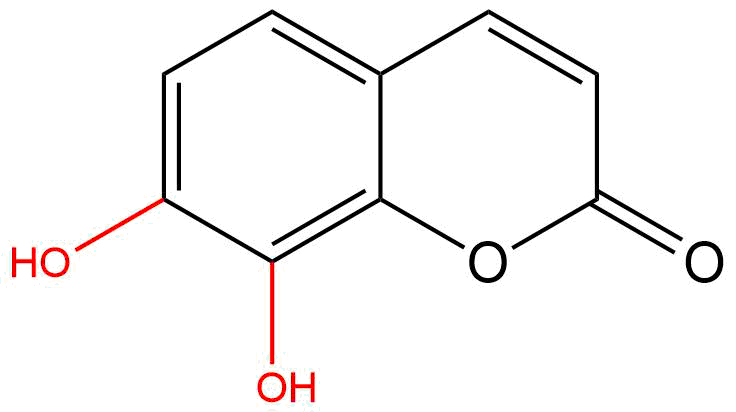 | Daphnetin | **0.64** | | **0.32** | | **5.12** | | >5.12 |
| 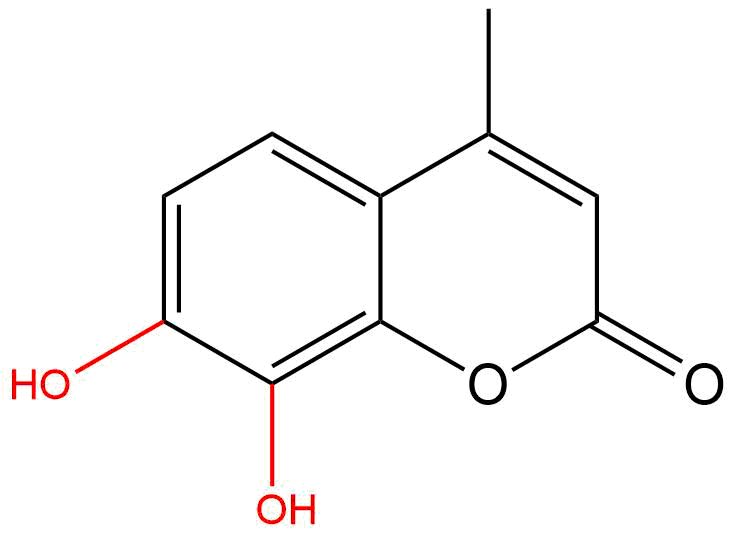 | 4-methyldaphnetin | **1.28** | | **1.28** | | **1.28** | | >5.12 |
| 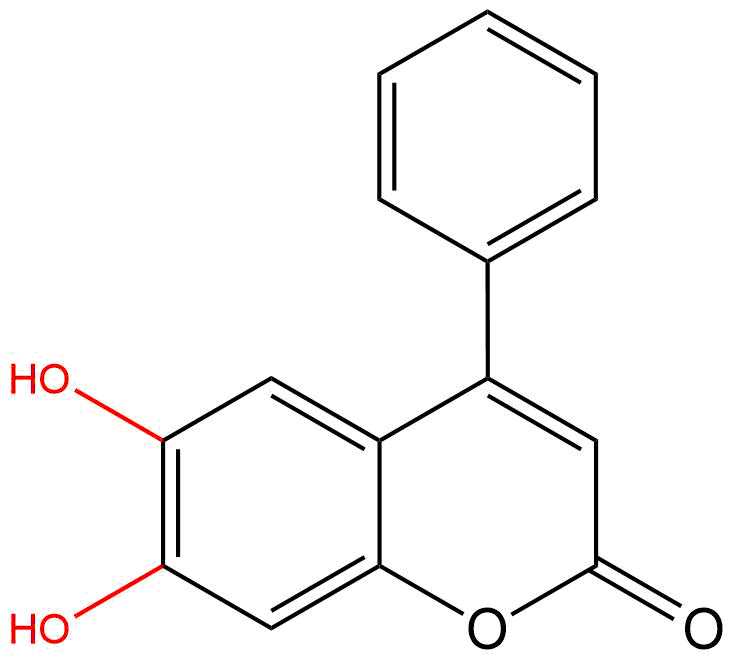 | 6,7-Dihydroxy-4-phenylcoumarin | **1.28** | | **1.28** | | **0.64** | | >5.12 |
| 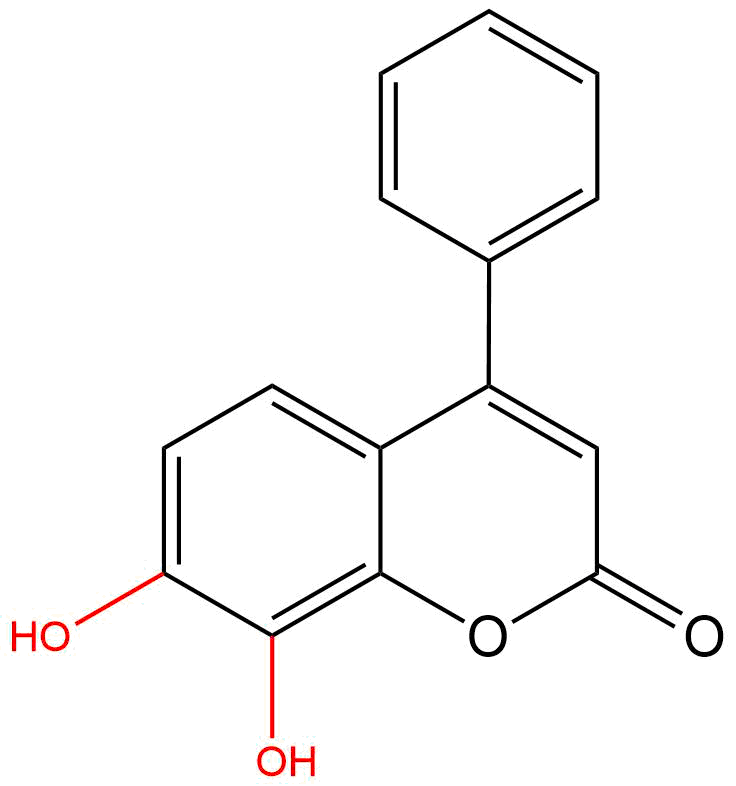 | 7,8-Dihydroxy-4-phenylcoumarin | **5.12** | | **2.56** | | **1.28** | | >5.12 |
| 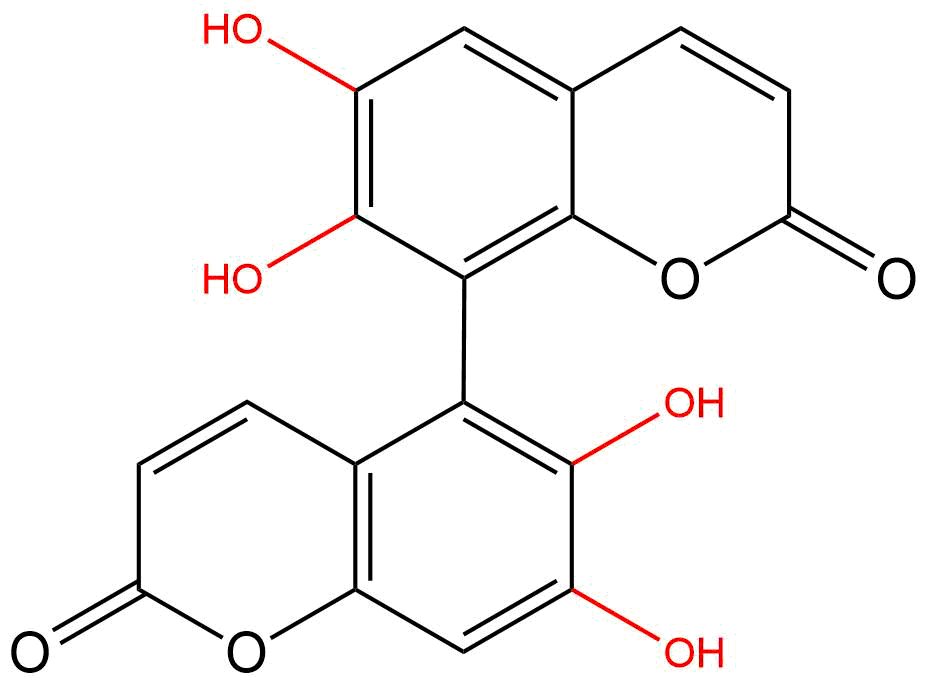 | Euphorbetin | >5.12 | | >5.12 | | >5.12 | | >5.12 |
| 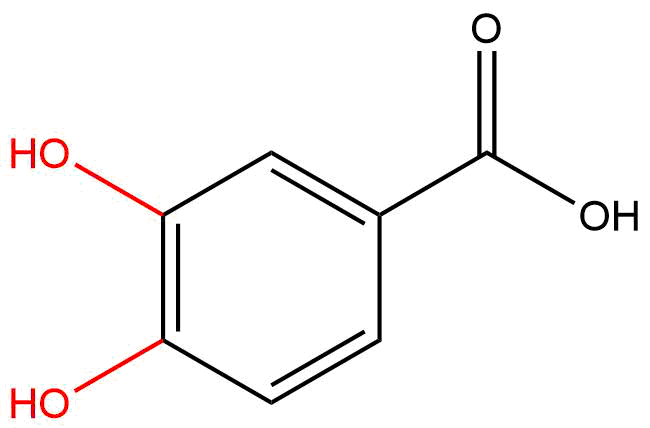 | Protocatechuic acid | **5.12** | | **5.12** | | **5.12** | | >5.12 |
| 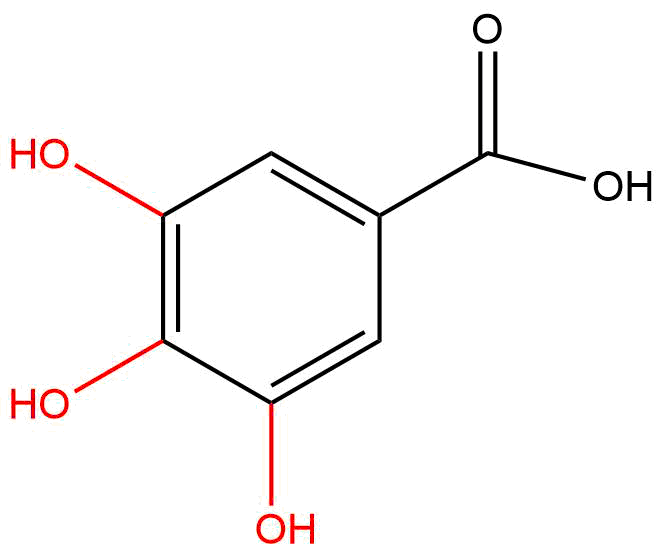 | Gallic acid | **5.12** | | **5.12** | | >5.12 | | >5.12 |
| 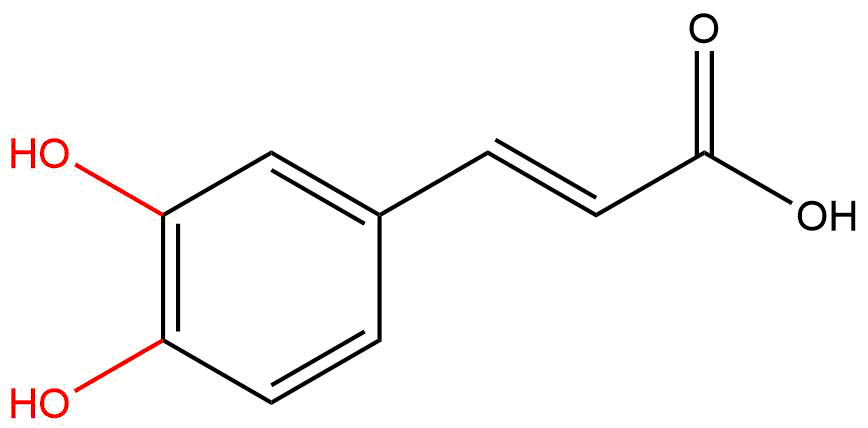 | Caffeic acid | **5.12** | | **5.12** | | >5.12 | | >5.12 |
| 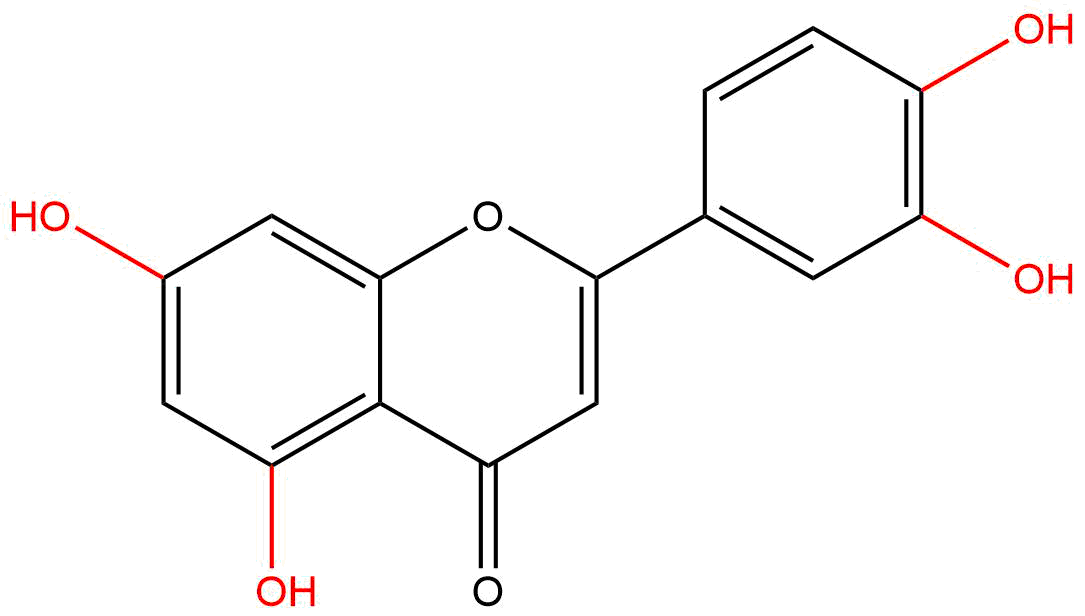 | Luteolin | >5.12 | | >5.12 | | >5.12 | | >5.12 |
| 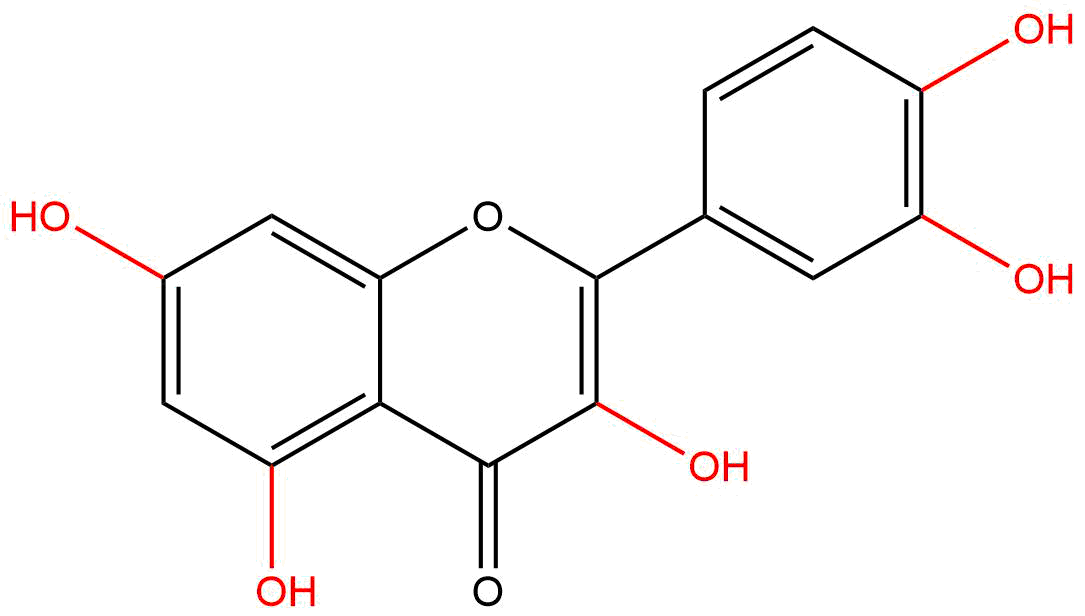 | Quercetin | >5.12 | | >5.12 | | >5.12 | | >5.12 |
| 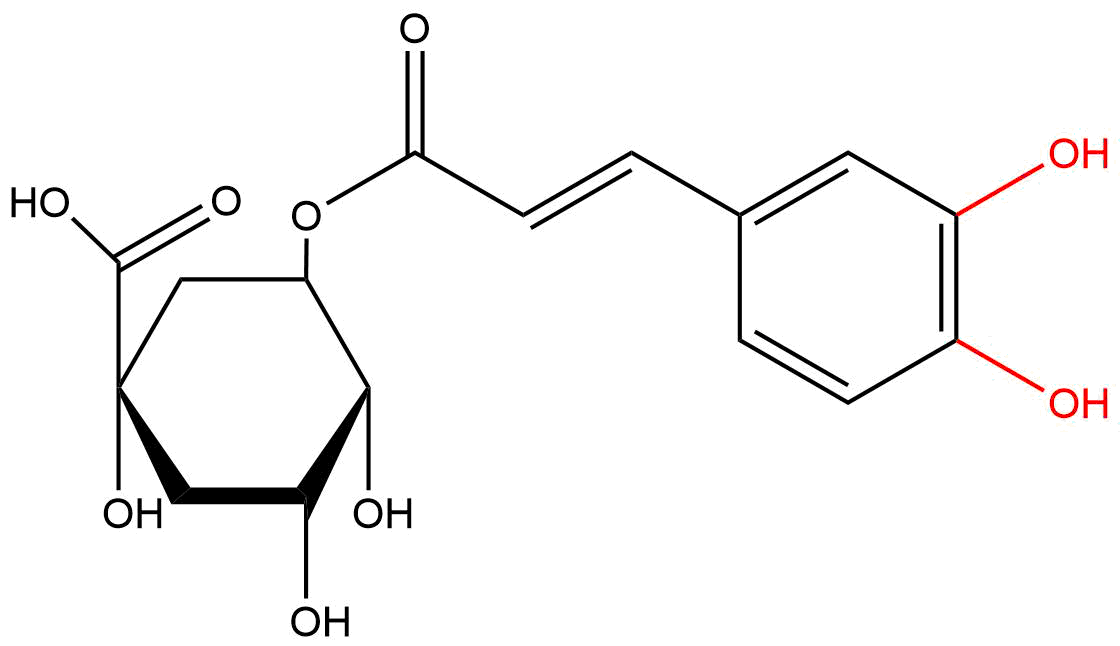 | Chlorogenic acid | >5.12 | | >5.12 | | **5.12** | | >5.12 |
| 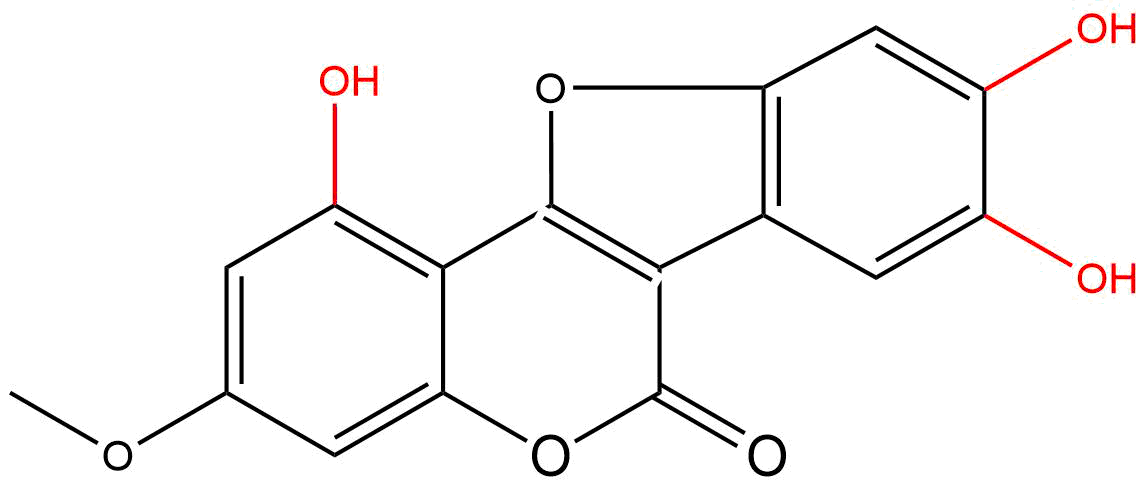 | Wedelolactone | >5.12 | | >5.12 | | >5.12 | | >5.12 |
| 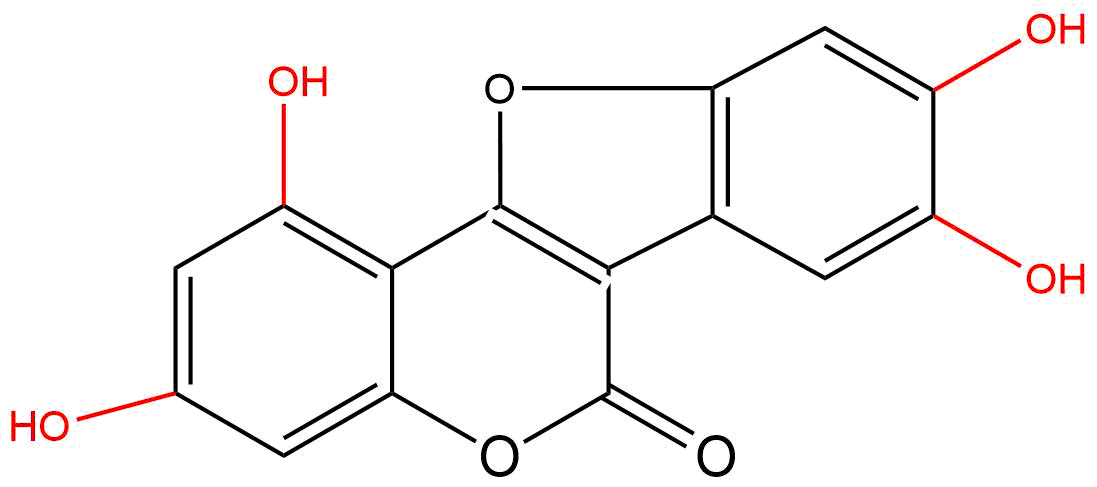 | Demethylwedelolactone | >5.12 | | >5.12 | | >5.12 | | >5.12 |
| 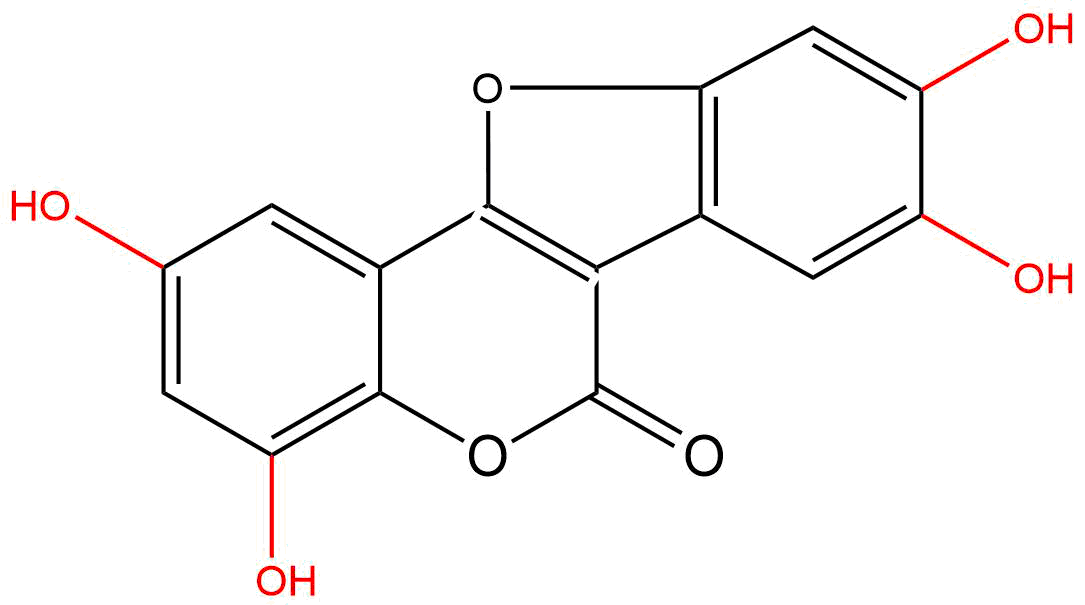 | Isodemethylwedelolactone | >5.12 | | >5.12 | | >5.12 | | >5.12 |
| 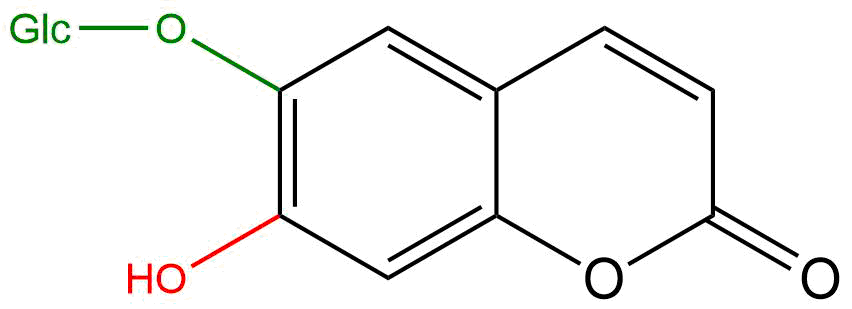 | Esculin | >5.12 | | >5.12 | | >5.12 | | >5.12 |
| 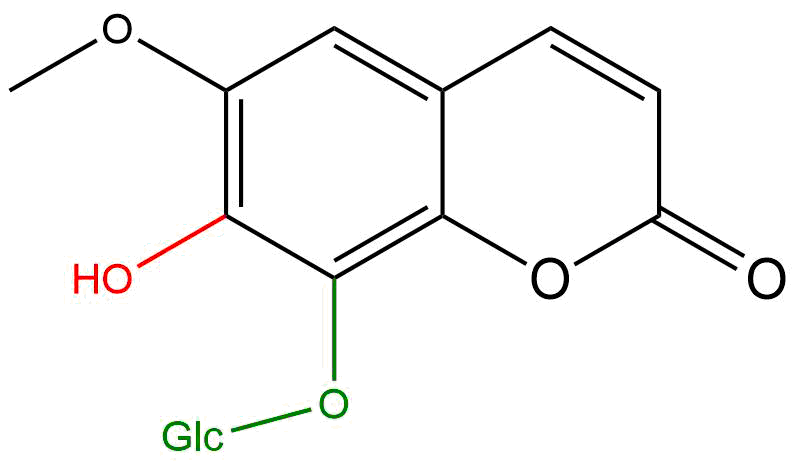 | Fraxin | >5.12 | | >5.12 | | >5.12 | | >5.12 |
| 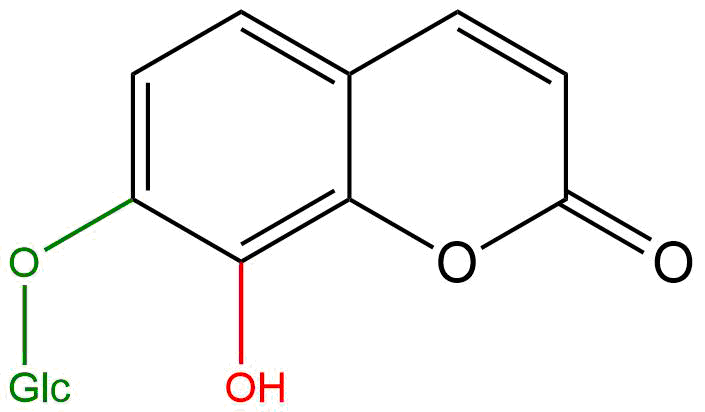 | Daphnin | >5.12 | | >5.12 | | >5.12 | | >5.12 |
| 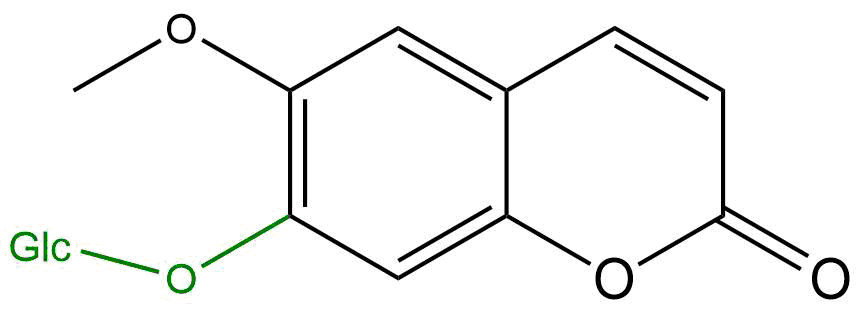 | Scopolin | >5.12 | | >5.12 | | >5.12 | | >5.12 |
| 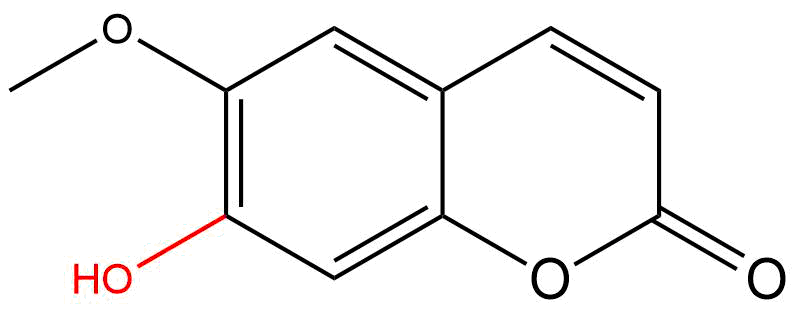 | Scopoletin | **5.12** | | **1.28** | | >5.12 | | >5.12 |
| 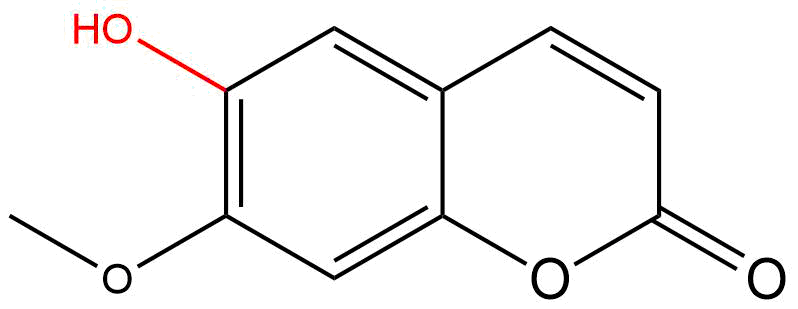 | Isoscopoletin | >5.12 | | >5.12 | | >5.12 | | >5.12 |
| 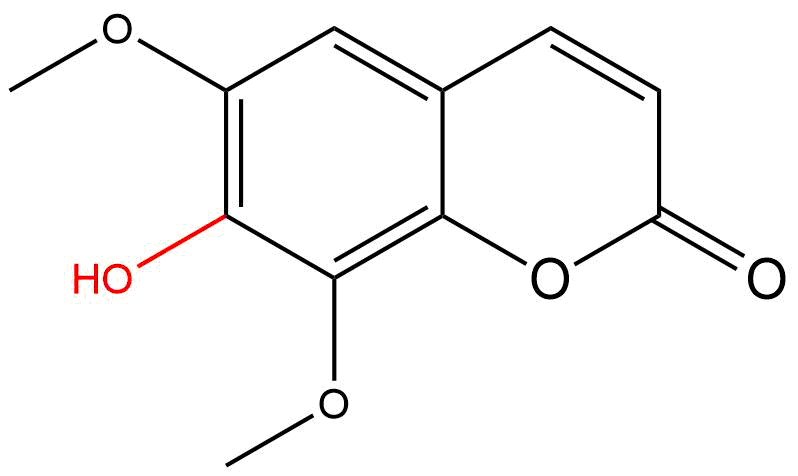 | Isofraxidin | >5.12 | | >5.12 | | **0.32** | | >5.12 |
| 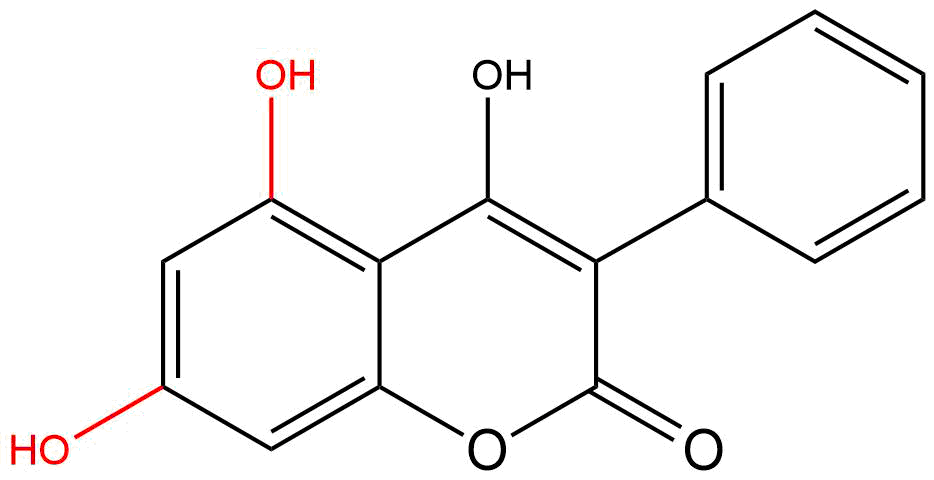 | 4,5,7-Trihydroxy-3-phenylcoumarin | >5.12 | | >5.12 | | **1.28** | | >5.12 |
| 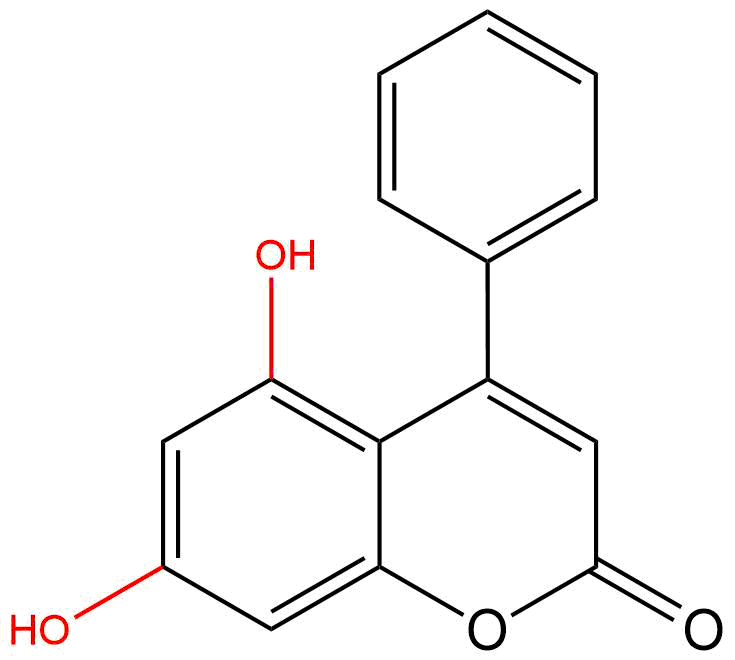 | 5,7-Dihydroxy-4-phenylcoumarin | >5.12 | | >5.12 | | >5.12 | | >5.12 |
| 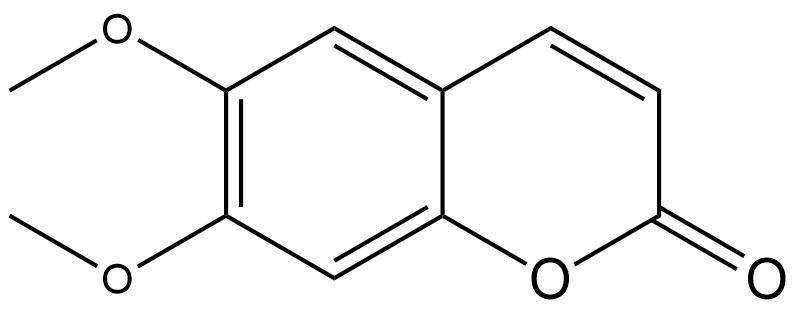 | Scoparone | >5.12 | | >5.12 | | >5.12 | | >5.12 |
| 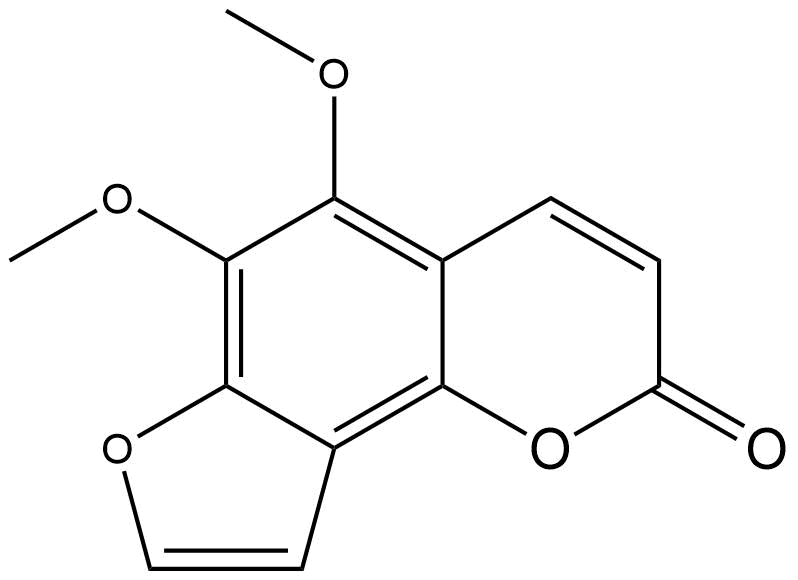 | Pimpinellin | >5.12 | | >5.12 | | >5.12 | | >5.12 |
| 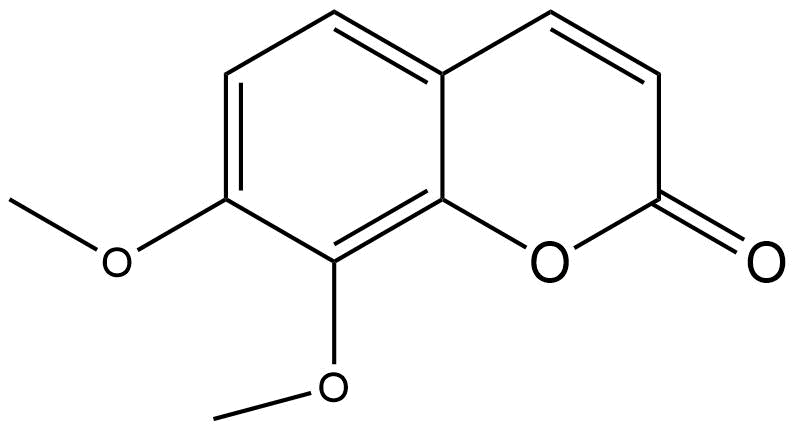 | Daphnetin dimethyl ether | >5.12 | | >5.12 | | >5.12 | | >5.12 |
| 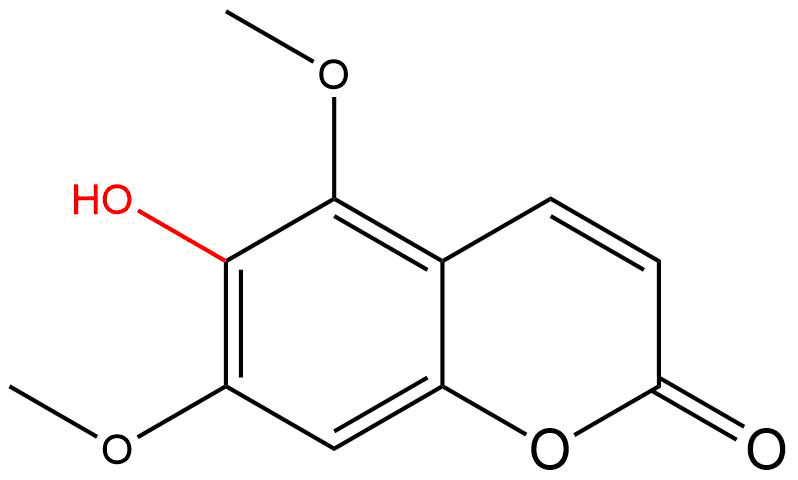 | Fraxinol | >5.12 | | >5.12 | | >5.12 | | >5.12 |
| 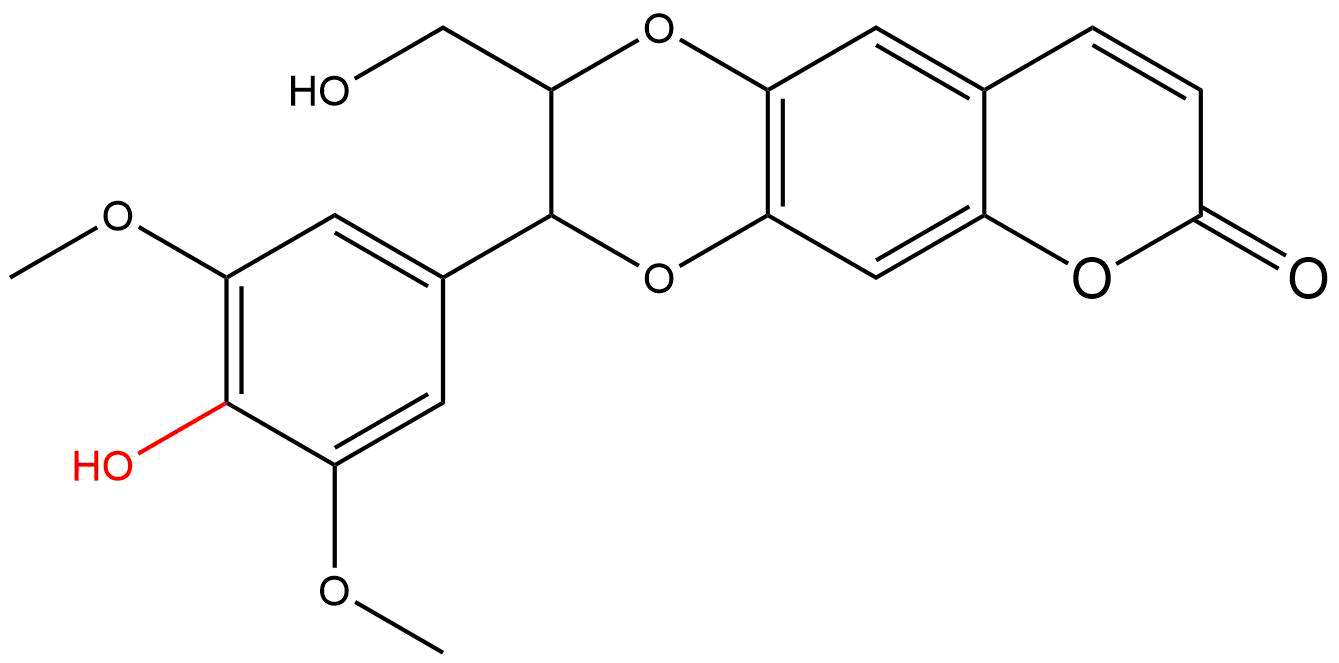 | Moluccanin | >5.12 | | >5.12 | | >5.12 | | >5.12 |

MIC, minimum inhibitory concentrations.

**Table S2. Bacterial strains used in this study.**

| Organism | Source |
| --- | --- |
| *Salmonella* Typhimurium ATCC 14028 | ATCC |
| *Salmonella* Typhimurium 15E475 | Guangzhou CDC |
| *Salmonella* Enteritidis ATCC 13076 | ATCC |
| *Salmonella* Braenderup H9812 | This study |
| *Enterococcus faecium* CAU 280 | This study |
| *Enterococcus faecium* CAU 361 | This study |
| *Enterococcus faecium* CAU 362 | This study |
| *Enterococcus faecium* CAU 363 | This study |
| *Enterococcus faecium* CAU 364 | This study |
| *Enterococcus faecium* CAU 365 | This study |
| *Enterococcus faecium* CAU 366 | This study |
| *Enterococcus faecium* CAU 367 | This study |
| *Enterococcus faecium* CAU 368 | This study |
| *Enterococcus faecium* CAU 369 | This study |
| *Enterococcus faecium* CAU 370 | This study |
| *Enterococcus faecium* CAU 371 | This study |
| *Enterococcus faecium* CAU 372 | This study |
| *Enterococcus faecium* CAU 373 | This study |
| *Enterococcus faecium* CAU 374 | This study |
| *Enterococcus faecium* CAU 375 | This study |
| *Enterococcus faecium* CAU 376 | This study |
| *Enterococcus faecium* CAU 377 | This study |
| *Enterococcus faecium* CAU 379 | This study |
| *Enterococcus faecium* CAU 380 | This study |
| *Enterococcus faecium* CAU 384 | This study |
| *Enterococcus faecium* CAU 385 | This study |
| *Enterococcus faecium* CAU 386 | This study |
| *Enterococcus faecium* CAU 387 | This study |
| *Enterococcus faecium* CAU 388 | This study |
| *Enterococcus faecium* CAU 389 | This study |
| *Enterococcus faecium* CAU 390 | This study |
| *Enterococcus faecium* CAU 391 | This study |
| *Enterococcus faecium* CAU 392 | This study |
| *Enterococcus faecium* CAU 393 | This study |
| *Enterococcus faecalis* ATCC 29212 | ATCC |
| *Enterococcus faecalis* JH2-2 | (Li *et al*, 2022) |
| *Enterococcus durans* 1-3 | This study |
| *Enterococcus avium* CAU 474 | This study |
| *Enterococcus casselifavus* CAU 472 | This study |
| *Staphylococcus aureus* ATCC 29213 | ATCC |
| *Staphylococcus aureus* T144 | (Liu *et al*, 2017) |
| *Staphylococcus chromogenes* 1N-1 | This study |
| *Bacillus subtilis* CAU21 | (Liu *et al*, 2017) |
| *Bacillus cereus* CAU45 | This study |
| *Klebsiella pneumoniae* WNX-1 | This study |
| *Pseudomonas aeruginosa* PAO-1 | This study |
| *Acinetobacter baumanni* ATCC 17978 | ATCC |
| *Aeromonas veroni* CVCC 3700 | CVCC |
| *Escherichia coli* ATCC 25922 | ATCC |
| *Escherichia coli* BL21(DE3) | This study |
| *Escherichia coli* B2 | (Song *et al*, 2020) |

ATCC, American type culture collection; Guangzhou CDC, Guangzhou center for disease control and prevention; CVCC, National center for veterinary culture collection.

**Table S3. Chemicals used in this study.**

| Chemicals | Source | Identifier |
| --- | --- | --- |
| Brain heart infusion broth (BHI) | Land Bridge Technology | Cat# CM917B |
| Lilly-Barnett medium (LB) | Land Bridge Technology | Cat# CM158 |
| M9 minimal medium (M9) | Coolaber | Cat# SL0060 |
| Mueller hinton broth (MHB) | Land Bridge Technology | Cat# CM901 |
| Tryptone soy broth (TSB) | Land Bridge Technology | Cat# CM301 |
| *Salmonella* chromogenic agar | Land Bridge Technology | Cat# ESM005 |
| *Enterococcus* agar | Land Bridge Technology | Cat# CM1528 |
| Glucose | Sigma-Aldrich | Cat# 50-99-7 |
| Agar | Sigma-Aldrich | Cat# 9002-18-0 |
| Crystal violet staining solution | Beyotime | Cat# C0121 |
| Streptomycin sulfate | Aladdin | Cat# 3810-74-0 |
| 2,2-Dipyridyl | Sigma-Aldrich | Cat# 366-18-7 |
| Iron (III) chloride hexahydrate | Sigma-Aldrich | Cat# 10025-77-1 |
| Esculetin | Sigma-Aldrich | Cat# PHL80449 |
| Fraxetin | Sigma-Aldrich | Cat# PHL89549 |
| Daphnetin | Sigma-Aldrich | Cat# PHL89621 |
| 4-methyldaphnetin | Sigma-Aldrich | Cat# 630748 |
| 6,7-Dihydroxy-4-phenylcoumarin | Sigma-Aldrich | Cat# 576441 |
| 7,8-Dihydroxy-4-phenylcoumarin | Sigma-Aldrich | Cat# 576441 |
| Euphorbetin | Biopurify | Cat# BP2128 |
| Protocatechuic acid | Biopurify | Cat# 03930590 |
| Gallic acid | Biopurify | Cat# BP0608 |
| Caffeic acid | Biopurify | Cat# C0625 |
| Luteolin | Biopurify | Cat# BP0896 |
| Quercetin | Biopurify | Cat# BP1187 |
| Chlorogenic acid | Biopurify | Cat# BP0345 |
| Wedelolactone | Biopurify | Cat# BP1451 |
| Demethylwedelolactone | Biopurify | Cat# BP0476 |
| Isodemethylwedelolactone | Biopurify | Cat# BP3117 |
| Esculin | Sigma-Aldrich | Cat# PHL89659 |
| Fraxin | Sigma-Aldrich | Cat# PHL89545 |
| Daphnin | Sigma-Aldrich | Cat# PHL85730 |
| Scopolin | Sigma-Aldrich | Cat# PHL82649 |
| Scopoletin | Sigma-Aldrich | Cat# PHL89516 |
| Isoscopoletin | Biopurify | Cat# BP3215 |
| Isofraxidin | Biopurify | Cat# BP0783 |
| 4,5,7-Trihydroxy-3-phenylcoumarin | Sigma-Aldrich | Cat# 578320 |
| 5,7-Dihydroxy-4-phenylcoumarin | Sigma-Aldrich | Cat# 576425 |
| Scoparone | Biopurify | Cat# BP1272 |
| Pimpinellin | Biopurify | Cat# BP1105 |
| Daphnetin dimethyl ether | Biopurify | Cat# BP3138 |
| Fraxinol | Biopurify | Cat# BP1580 |
| Moluccanin | Biopurify | Cat# BP1788 |

**Table S4. Primers used in this study.**

| Target | Primers | Function | Reference |
| --- | --- | --- | --- |
| *rpoD*-F | GTGAAATGGGCACTGTTGAACTG | The detection of virulence gene expression | (Zhang *et al*, 2020) |
| *rpoD*-R | TTCCAGCAGATAGGTAATGGCTTC |  |  |
| *hilA*-F | TGTCGGAAGATAAAGAGCAT |  |  |
| *hilA*-R | AAGGAAGTATCGCCAATGTA |  |  |
| *invA*-F | GAAATTATCGCCACGTTCGGGCAA |  |  |
| *invA*-R | TCATCGCACCGTCAAAGGAACC |  |  |
| *sopD*-F | ATTAATGCCGGTAACTTTGA |  |  |
| *sopD*-R | CTCTGAAAACGGTGAATAGC |  |  |
| *invF*-F | GCAGGATTAGTGGACACGAC | The detection of virulence gene expression | (Choi *et al*, 2007) |
| *invF*-R | TTTACGATCTTGCCAAATAGCG |  |  |
| *sigD*-F | AACCGTTCTGGGTAAACAAGAC |  |  |
| *sigD*-R | GGTCCGCTTTAACTTTGGCTAAC |  |  |
| *sicA*-F | ATTTGGGATGCCGTTAGTGAAG |  |  |
| *sicA*-R | TAAACCGTCCATCATATCTTGAGG |  |  |
| *sipB*-F | GCCGATGAAATTGTGAAGGC | The detection of virulence gene expression | This study |
| *sipB*-R | CCTAATCCTTCCAGCGCTTT |  |  |
| 16S rRNA-F | CGGTGAATACGTTCYCGG | Transformants check (Reference gene) | This study |
| 16S rRNA-R | GGWTACCTTGTTACGACTT |  |  |
| *cat*-F | ATGAACTTTAATAAAATT | Transformants check (Resistant marker) |  |
| *cat*-R | TTATAAAAGCCAGTCATT |  |  |
| *cat* ^pro^-F | ccgccccgttcgtaagccatTGATCGGCACGTAAGAGGT | Construction of the *bgl*-overexpression plasmid | This study |
| *cat* ^pro^-R | TTTAGCTTCCTTAGCTCCTGAAAAT |  |  |
| *cat* ^pro^::*bgl*-F | caggagctaaggaagctaaaATGTCAATTTTGAAAAATGATT |  |  |
| *cat* ^pro^::*bgl*-R | ttcgactgcggcgagcggaaTTATAATTCTTCTCCATTCGTA |  |  |
| pET-28a-*bgl* NcoI-F | TAAGAAGGAGATATACCATGGATATGTCAATTTTGAAAAATG | Construction of the prokaryotic expression plasmid | This study |
| pET-28a-*bgl* XhoI-R | GTGGTGGTGGTGGTGCTCGAGTAATTCTTCTCCATTCGTA |  |  |

**Table S5. Mass conditions of fraxetin (FXE) and fraxin (FX).**

|  | Channel  (Ch) | Precursor ion  (m/ z) | | Daughter ion  (m/ z) | | Retention time  (s) | | Q1 Pre Bias (V) | CE (V) | Q3 Pre Bias(V) |  |
| --- | --- | --- | --- | --- | --- | --- | --- | --- | --- | --- | --- |
| Fraxetin | Ch1 | 207.20 | | 192.25 | | 228.60 | | 15.00 | 14.00 | 18.00 |  |
|  | Ch2 | 207.20 | | 108.15 | | 228.60 | | 15.00 | 21.00 | 16.00 |  |
|  | Ch3 | 207.20 | | 164.20 | | 228.60 | | 15.00 | 23.00 | 30.00 |  |
| Fraxin | | Ch1 | | 369.30 | | 207.25 | | 214.10 | 17.00 | 19.00 | 19.00 |
|  |  | Ch2 | | 369.30 | | 192.20 | | 214.10 | 16.00 | 31.00 | 17.00 |
|  |  | Ch3 | | 369.30 | | 108.10 | | 214.10 | 16.00 | 43.00 | 19.00 |
|  |  | Ch4 | | 369.30 | | 163.15 | | 214.10 | 17.00 | 44.00 | 29.00 |

**
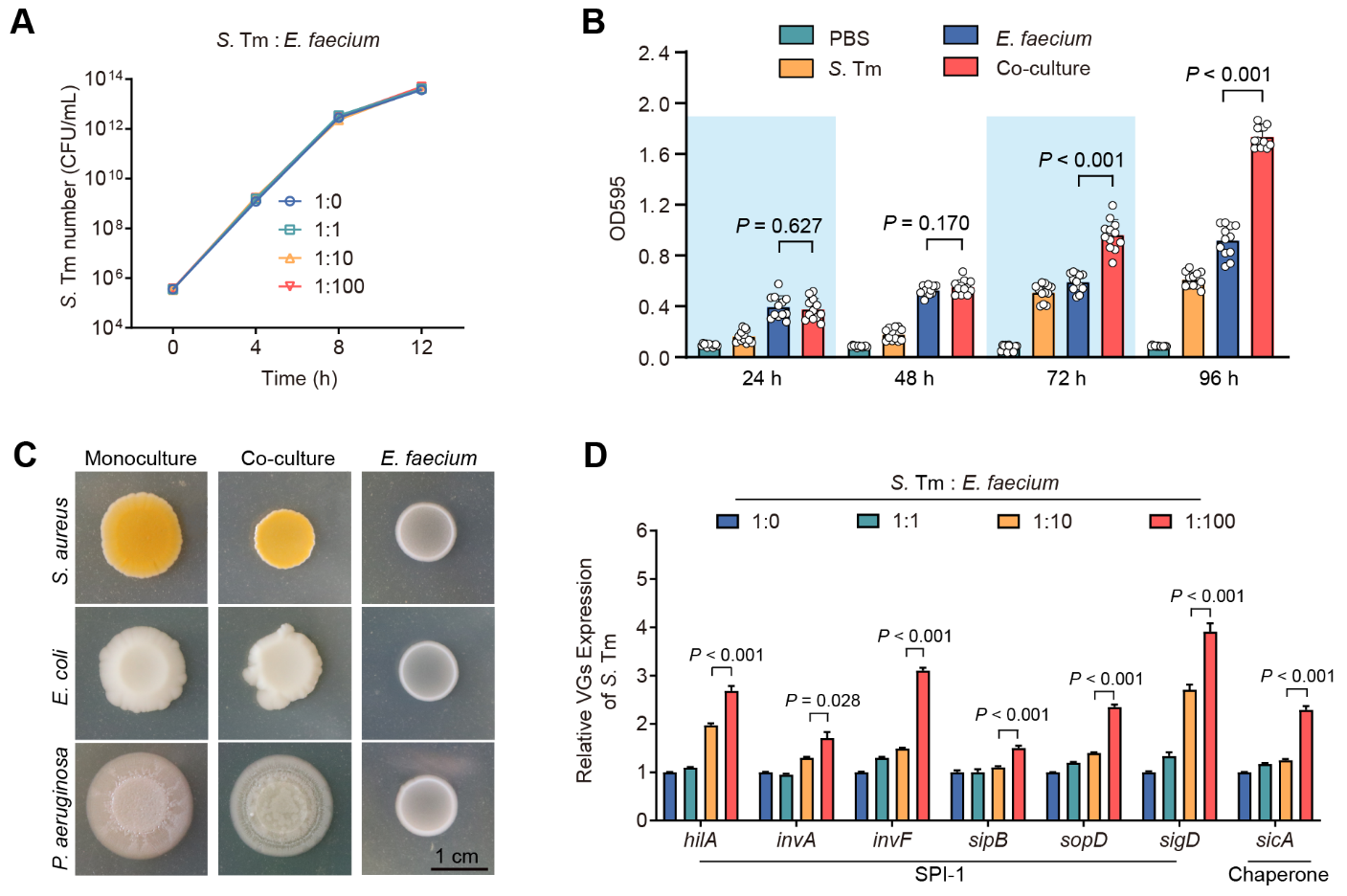
**

**Fig. S1. Enterococci promote the growth and pathogenicity of *S*. Tm.**

(A) Enumeration of *S*. Tm 15E475 in the presence of *E. faecium* CAU369 at different inoculum ratios under planktonic culture conditions. *n* = 3 biological replicates with three technical replicates. (B) Quantification of biofilm biomass formed by *E. faecium* CAU369 and *S*. Tm 15E475 using the standard crystal violet assay. *n* = 12 biological replicates with three technical replicates. (C) Morphological characterization of co-cultured macrocolonies comprising pathogenic bacteria and *E. faecium* CAU369 (at a ratio of 1: 100) in comparision with single-species macrocolonies. (D) Relative virulence genes (VGs) expression of *S*. Tm 15E475 at different inoculum ratios. *n* = 3 biological replicates with three technical replicates. Results represent the mean ± standard error. *P*-values were calculated using the independent samples *t*-test.


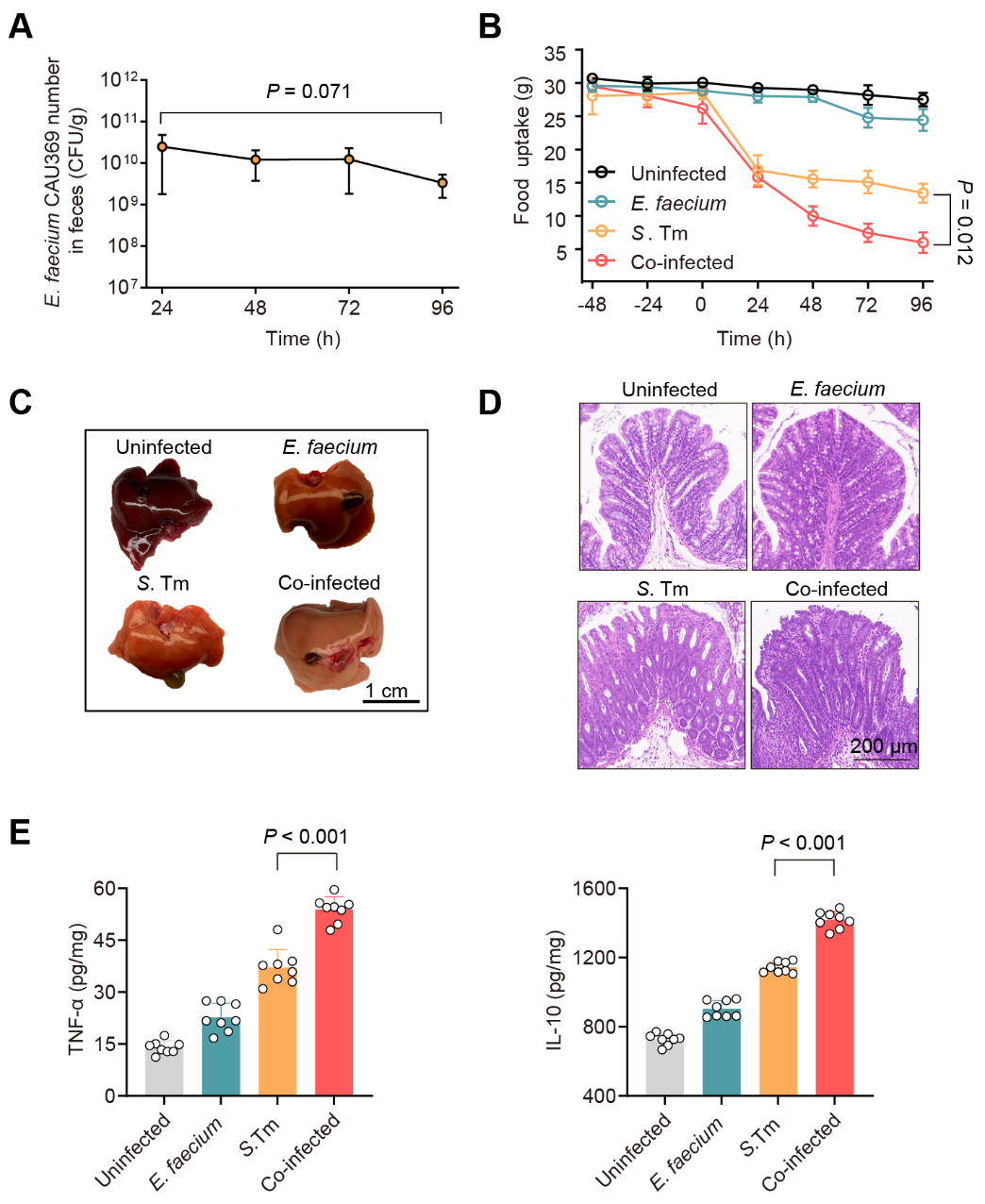


**Fig. S2. Enterococci aggravate the severity of *S*. Tm-induced diseases.**

(A) Bacterial loads of *E. faecium* CAU369 in feces. Mice were given 1×10^9^ CFUs of *E. faecium* CAU369 by oral gavage. (B) Food uptake of uninfected, mono- or co-infected mice. Data at each time point represents the total amount of food consumed by the corresponding group of mice. (C and D) Phenotype of livers (C) and hematoxylin and eosin (H&E) staining images of colon (D) from uninfected, mono- or co-infected mice. (E) Levels of TNF-α and IL-10 in livers. Results represent the mean ± standard error. *P*-values were calculated using the independent samples *t*-test.


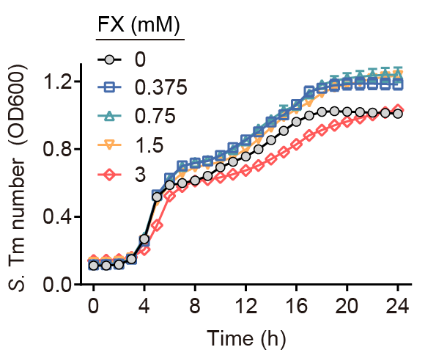


**Fig. S3. Growth curves of *S*. Tm 15E475 with the addition of FX for 24 h.**


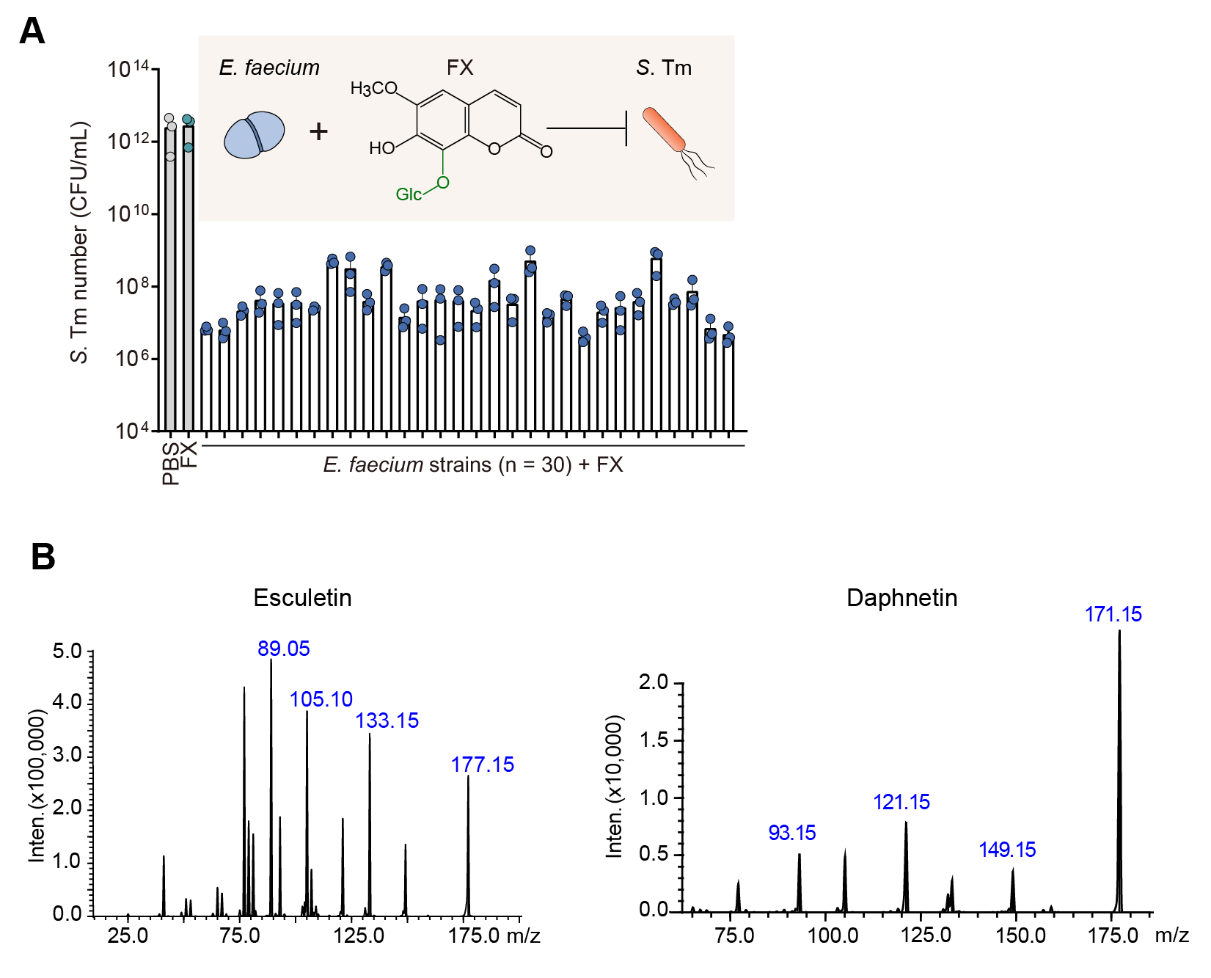


**Fig. S4. Enterococci hydrolyze diverse glucosides to bioactive aglycones.**

(A) Enumeration of *S*. Tm 15E475 in co-cultures with FX or various *E. faecium* isolates plus FX, at 12 h. (B) Mass spectrometry/mass spectrometry (MS/MS) spectra of esculetin and daphnetin, respectively.


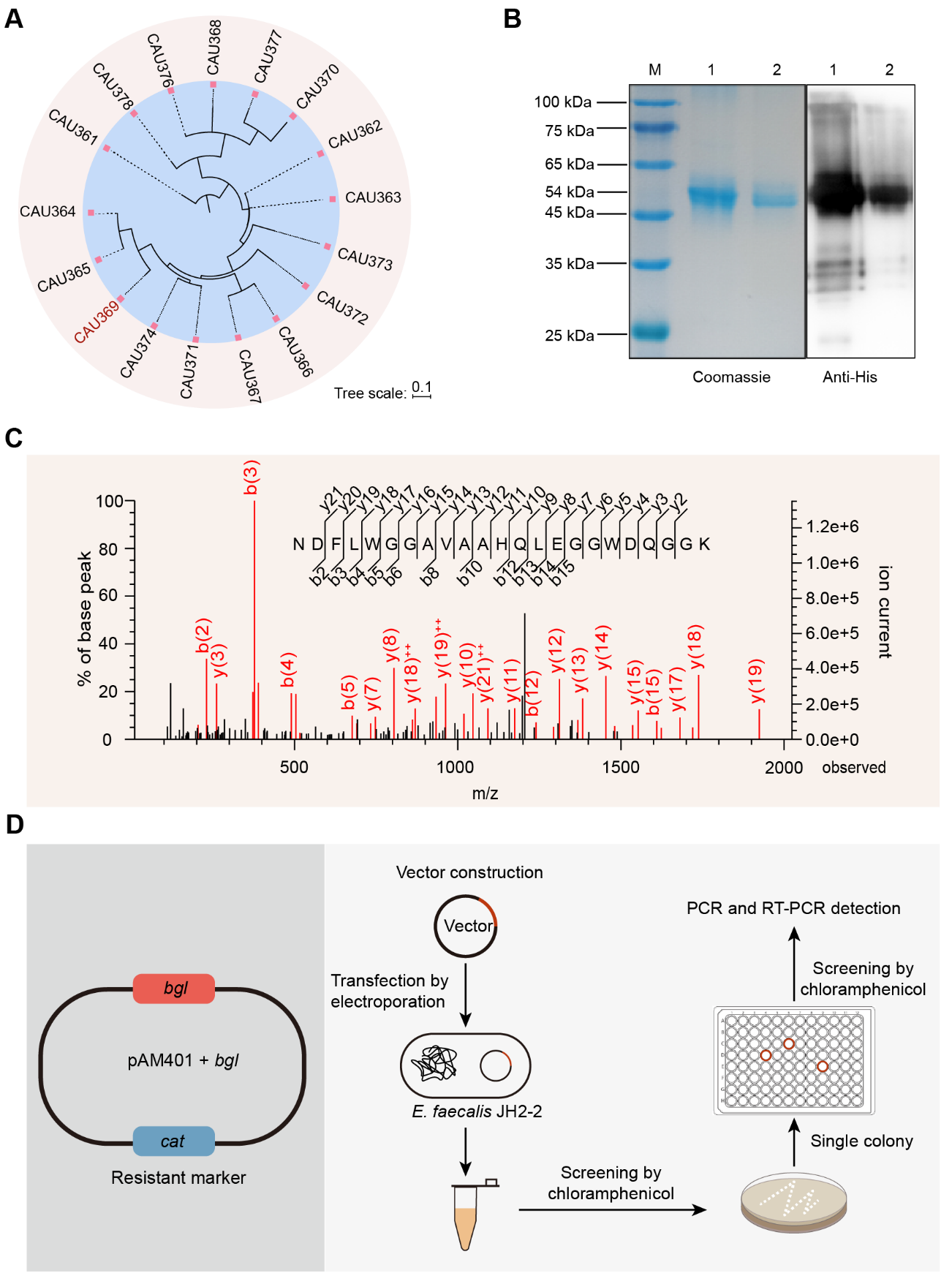


**Fig. S5. Enterococci hydrolyze coumarin glycosides via BGLs.**

(A) The phylogenetic tree of diverse *E. faecium* isolates carrying with the *bgl* gene (*n* = 17). (B) Purification of the BGL-His protein from *E. faecium* CAU369. BGL proteins were subjected to SDS-PAGE analysis with Coomassie Blue staining (left panel) and western blot analysis using anti-His antibody (right panel). M, marker; 1, eluting with 100 mM imidazole in buffer A; 2, eluting with 500 mM imidazole in buffer A. The size for the BGL-His protein is approximately 54 kDa. (C) Sequences of the unique fragments of purified BGL using the fragment ions generated from MALDI-TOF/TOF-MS. (D) Vector construction and transfection scheme of electroporation.


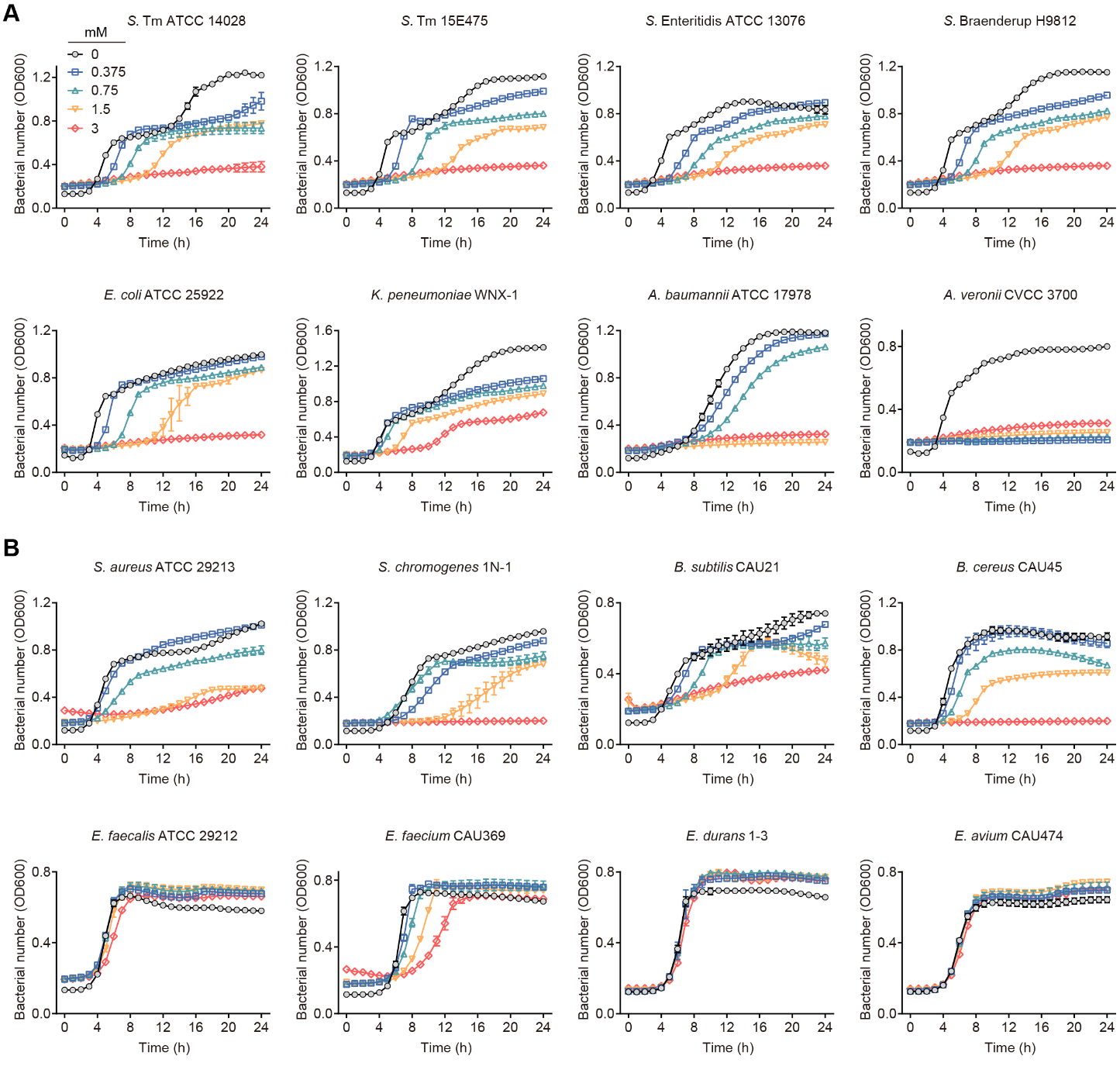


**Fig. S6. FXE inhibits a variety of pathogenic bacteria except enterococci.**

(A) Growth curves of Gram-negative bacteria with gradient concentrations of FXE for 24 h. *n* = 3 biological replicates with three technical replicates. (B) Growth curves of Gram-positive bacteria with gradient concentrations of FXE for 24 h. *n* = 3 biological replicates with three technical replicates.

**
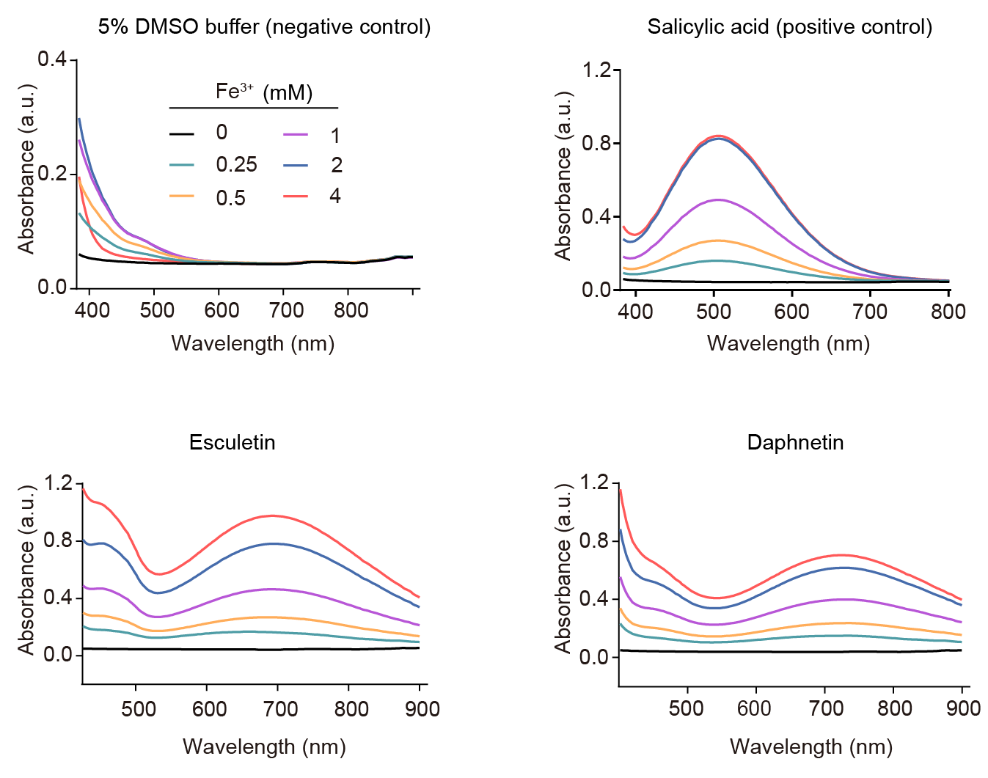
**

**Fig. S7. Catechol coumarin aglycones chelate irons (III).**

UV−Vis spectra of gradient concentrations of iron (III) with 1 mM salicylic acid, esculetin and daphnetin, respectively, in 5% DMSO buffer.


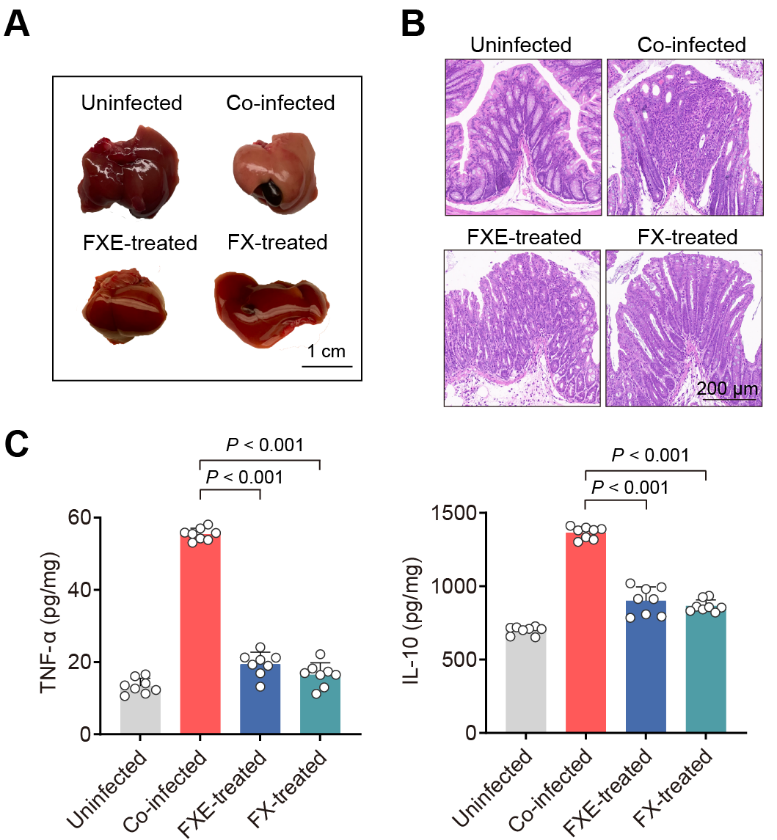


**Fig. S8. Coumarin glycosides reverse the driving role of enterococci *in vivo*.**

(A and B) Phenotype of livers (A) and hematoxylin and eosin (H&E) staining images of colon (B) from uninfected, co-infected and FXE/FX-treated mice. (C) Levels of TNF-α and IL-10 in livers. Results represent the mean ± standard error. *P*-values were calculated using one-way analysis of variance (ANOVA) with the LSD post hoc test.

**Reference**

Choi J, Shin D, Ryu S. Implication of quorum sensing in *Salmonella* enterica serovar typhimurium virulence: the *luxS* gene is necessary for expression of genes in pathogenicity island 1. *Infect Immun*. 2007;75(10):4885-90.

Li Q, Chen S, Zhu K, Huang X, Huang Y, Shen Z, Ding S, Gu D, Yang Q, Sun H, et al. Collateral sensitivity to pleuromutilins in vancomycin-resistant *Enterococcus faecium*. *Nat Commun.* 2022;13(1):1888.

Liu Y, Ding S, Dietrich R, Märtlbauer E, Zhu K. A biosurfactant-inspired heptapeptide with improved specificity to kill MRSA. *Angew Chem Int Ed Engl.* 2017;56(6):1486-1490.

Song M, Liu Y, Huang X, Ding S, Wang Y, Shen J, Zhu K. A broad-spectrum antibiotic adjuvant reverses multidrug-resistant Gram-negative pathogens. *Nat Microbiol.* 2020;5(8):1040-1050.

Zhang JS, Corredig M, Morales-Rayas R, Hassan A, Griffiths MW, LaPointe G. Downregulation of *Salmonella* virulence gene expression during invasion of epithelial cells treated with *Lactococcus lactis* subsp. cremoris JFR1 Requires OppA. *Probiotics Antimicro proteins.* 2020;12(2):577-588.
