# Supplementary figures and images for: Coumarin Glycosides Reverse Enterococci-Facilitated Enteric Infections
Source: Research (Wash D C). 2024 May 16;7:0374. doi: 10.34133/research.0374 (PMC11096794; doi:10.34133/research.0374)

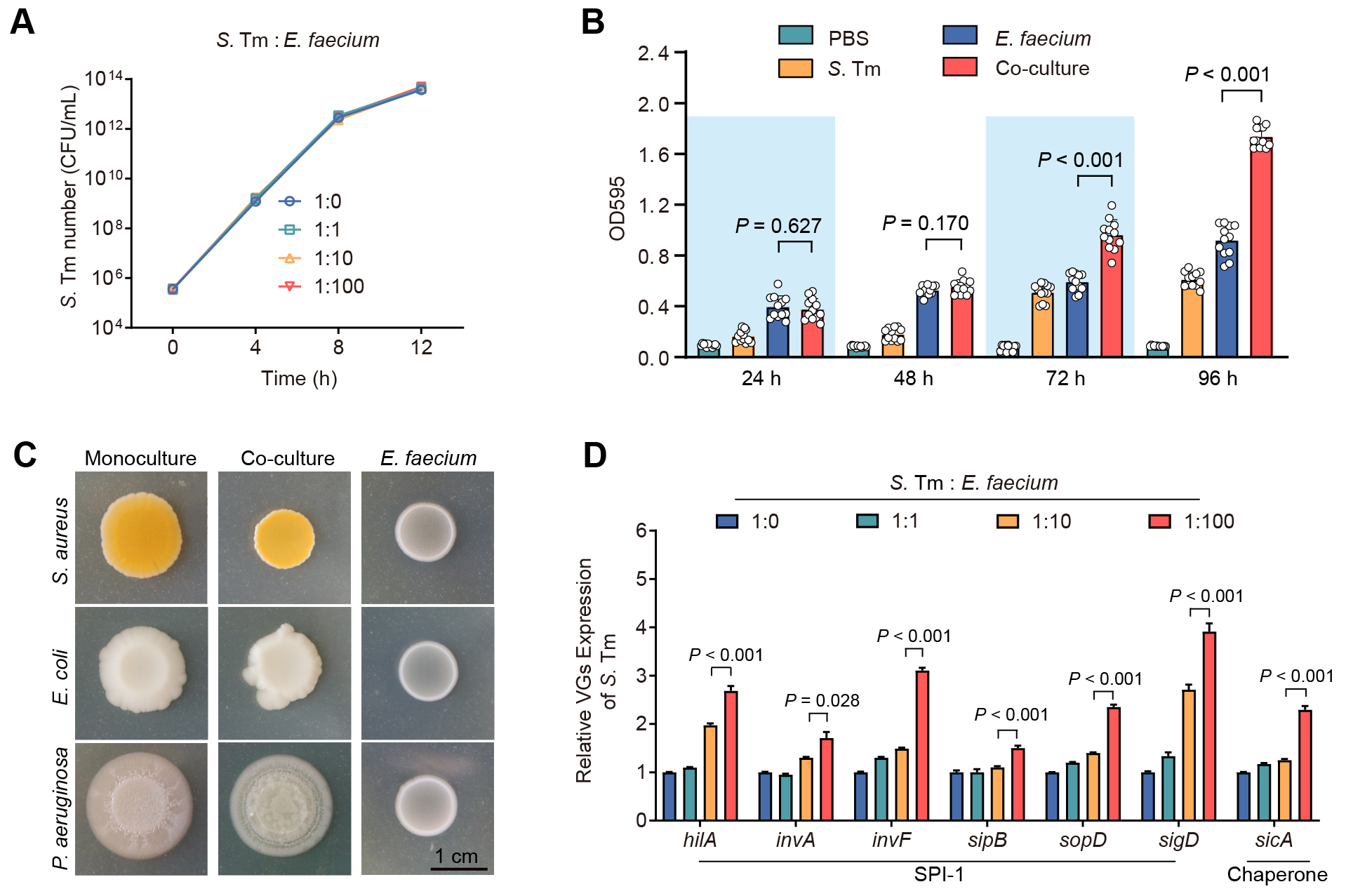

Supplement: Supplementary 1 — Figs. S1 to S8 Tables S1 to S5 [file research.0374.f1.zip › Figure S1.tif]

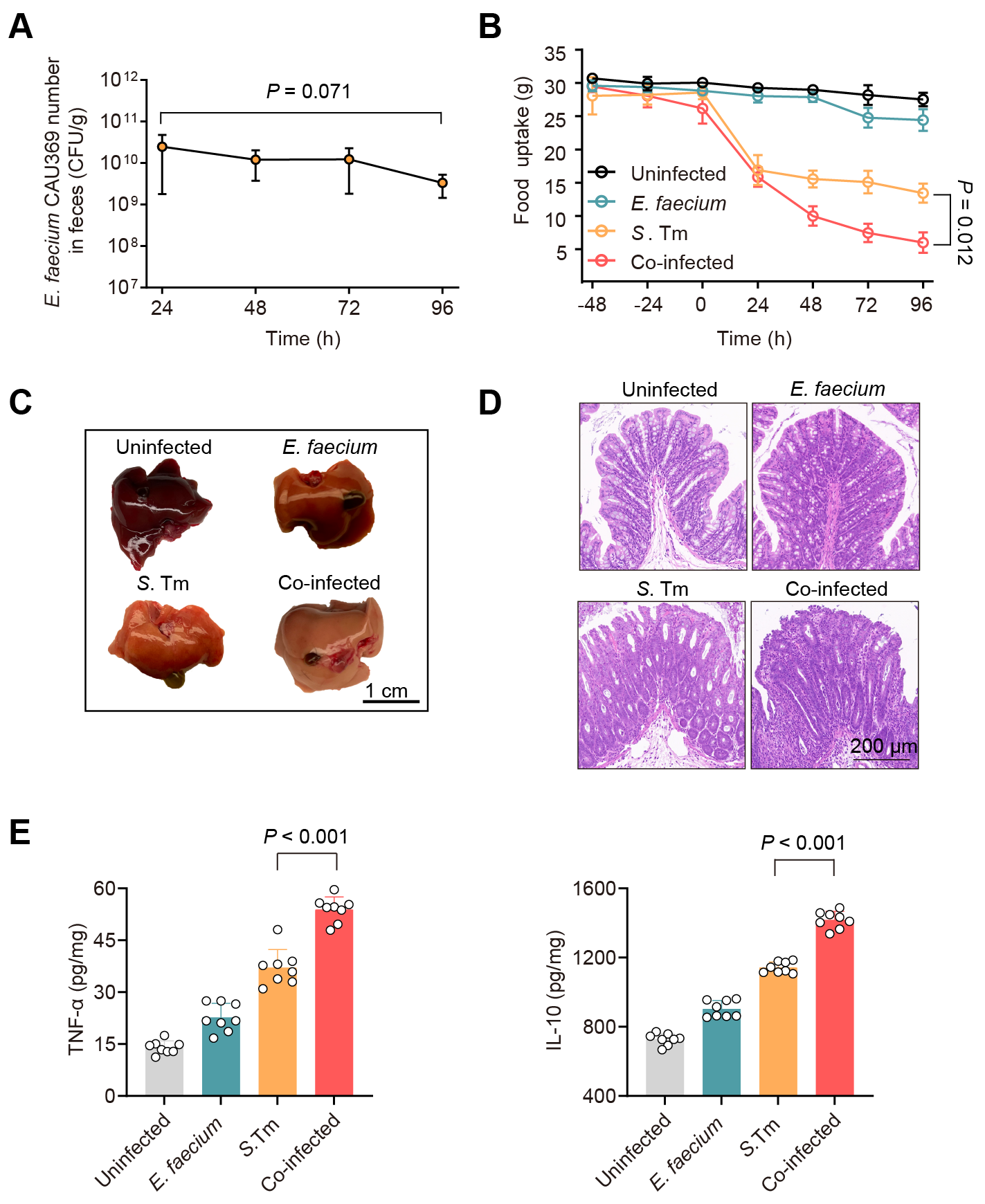

Supplement: Supplementary 1 — Figs. S1 to S8 Tables S1 to S5 [file research.0374.f1.zip › Figure S2.tif]

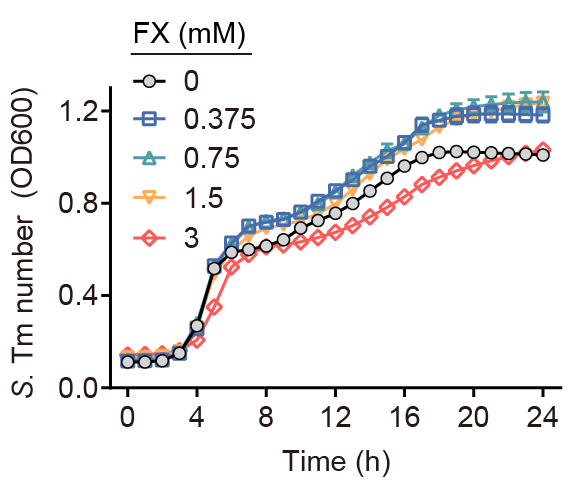

Supplement: Supplementary 1 — Figs. S1 to S8 Tables S1 to S5 [file research.0374.f1.zip › Figure S3.tif]

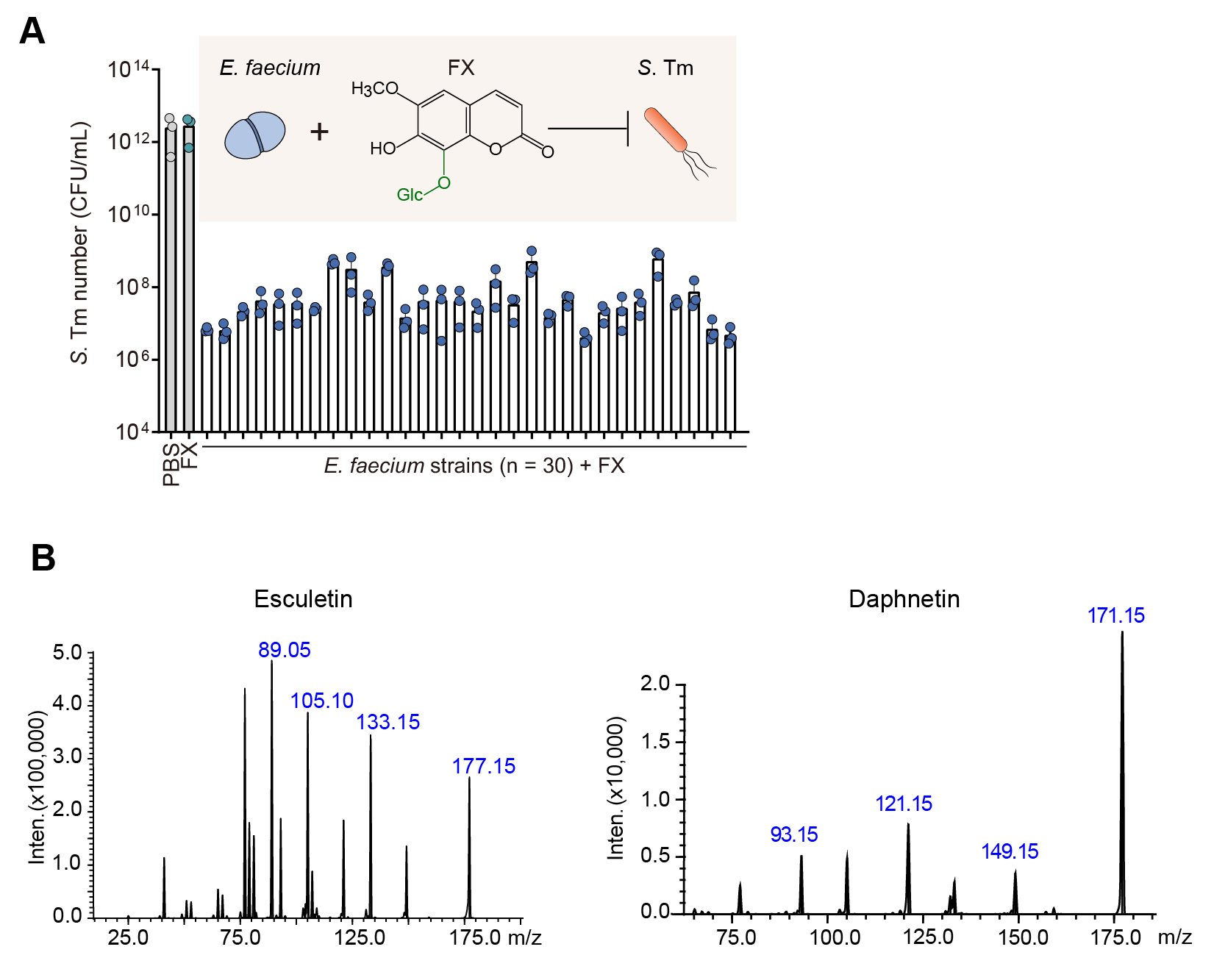

Supplement: Supplementary 1 — Figs. S1 to S8 Tables S1 to S5 [file research.0374.f1.zip › Figure S4.tif]

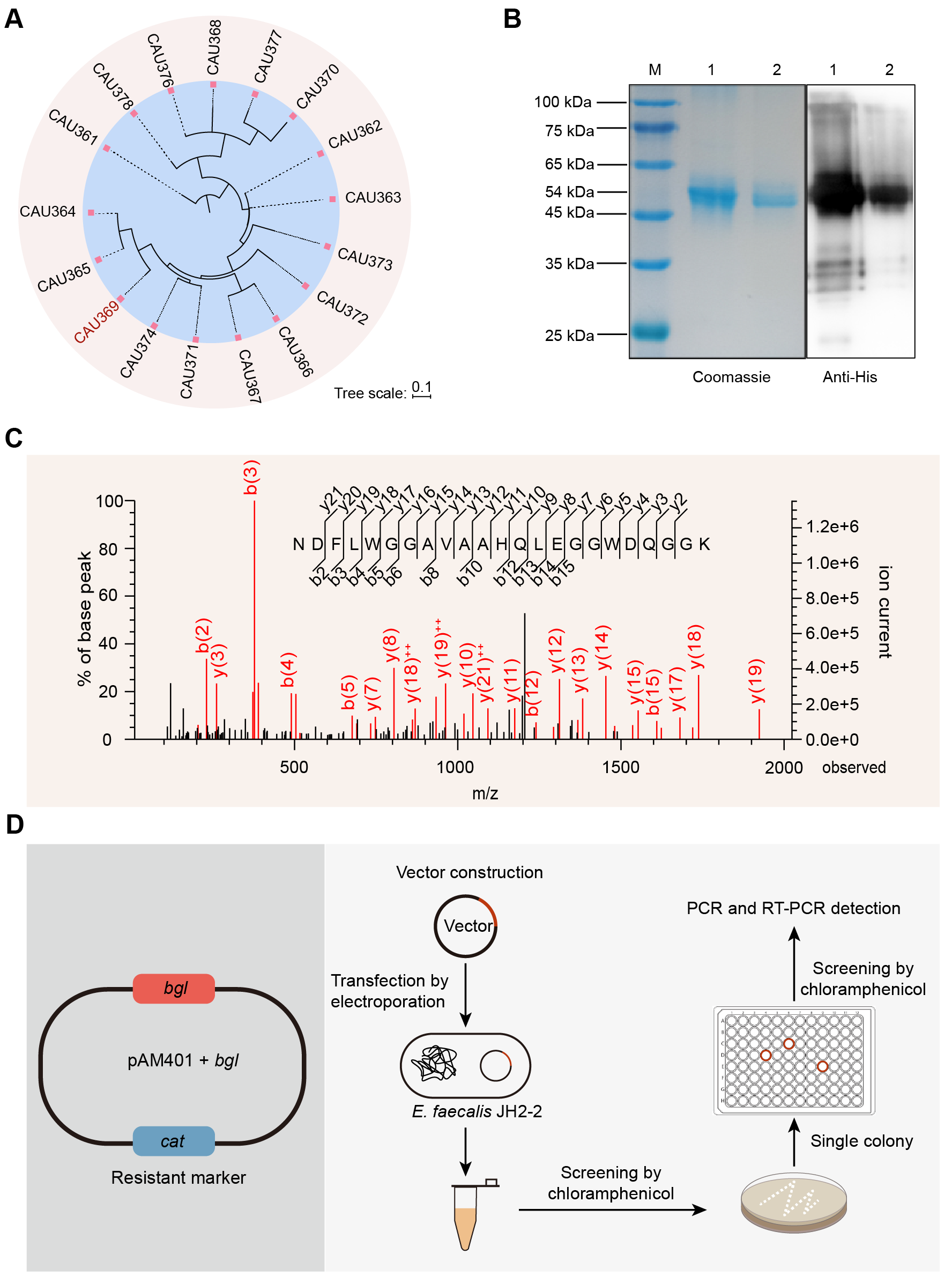

Supplement: Supplementary 1 — Figs. S1 to S8 Tables S1 to S5 [file research.0374.f1.zip › Figure S5.tif]

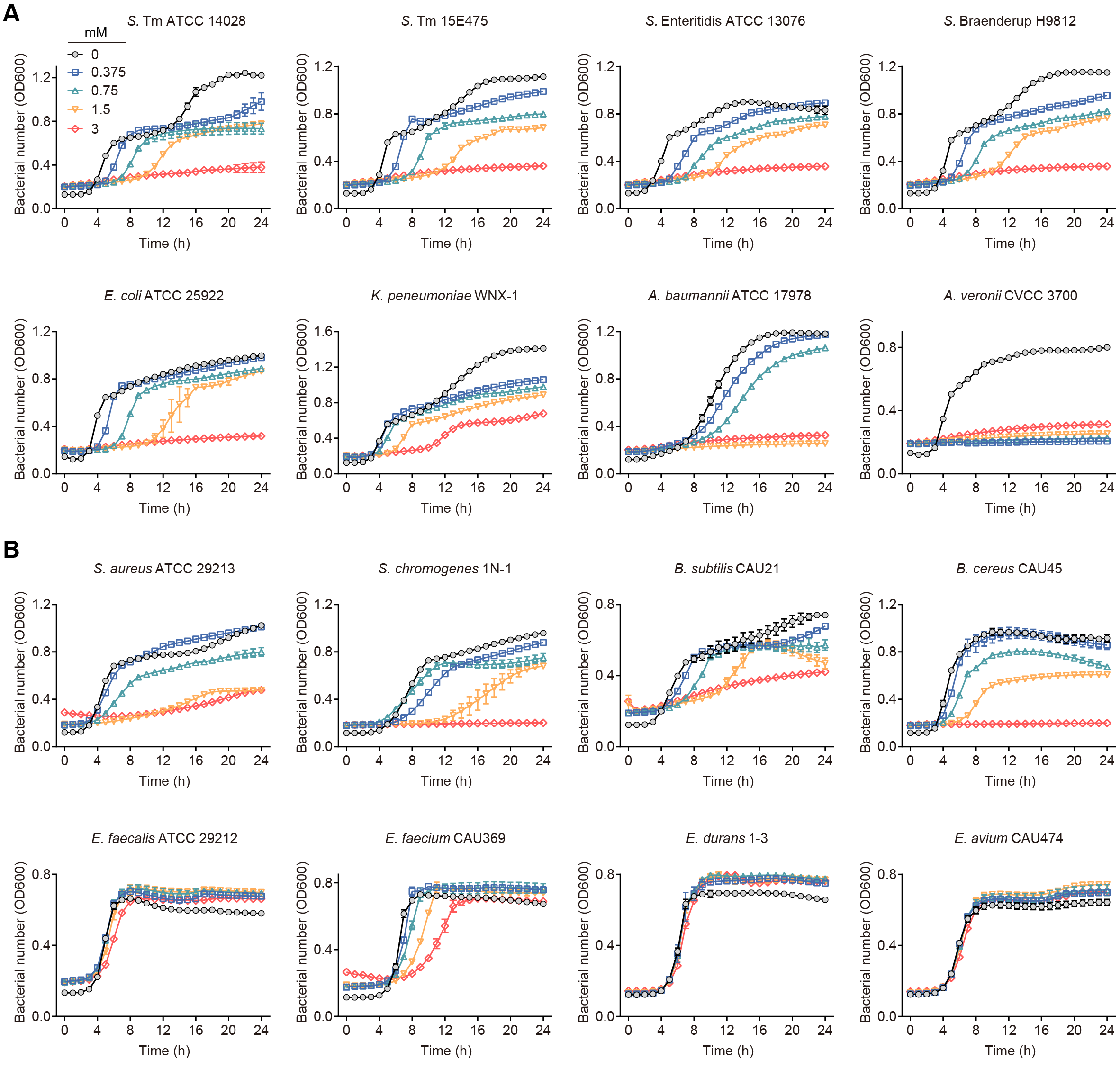

Supplement: Supplementary 1 — Figs. S1 to S8 Tables S1 to S5 [file research.0374.f1.zip › Figure S6.tif]

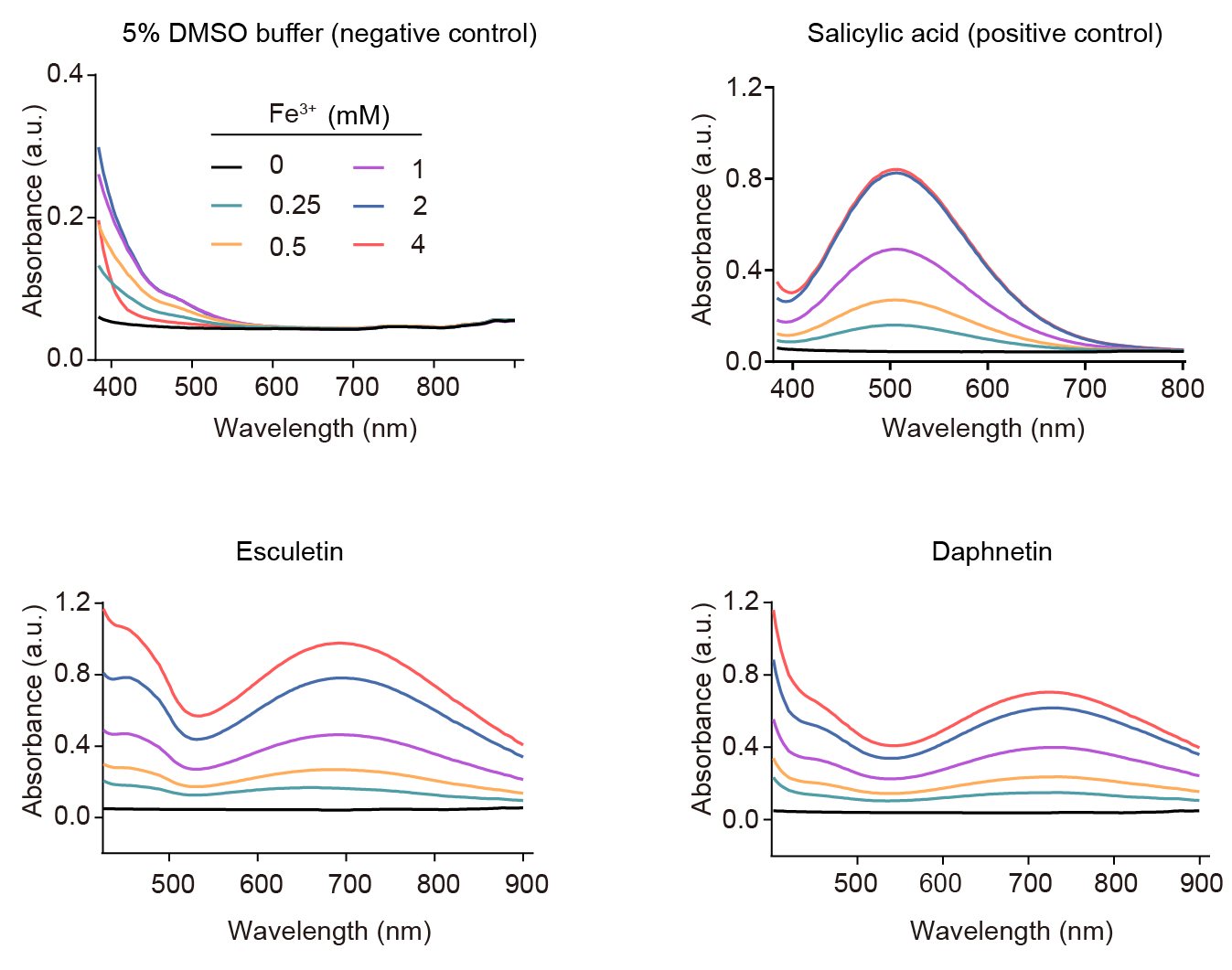

Supplement: Supplementary 1 — Figs. S1 to S8 Tables S1 to S5 [file research.0374.f1.zip › Figure S7.tif]

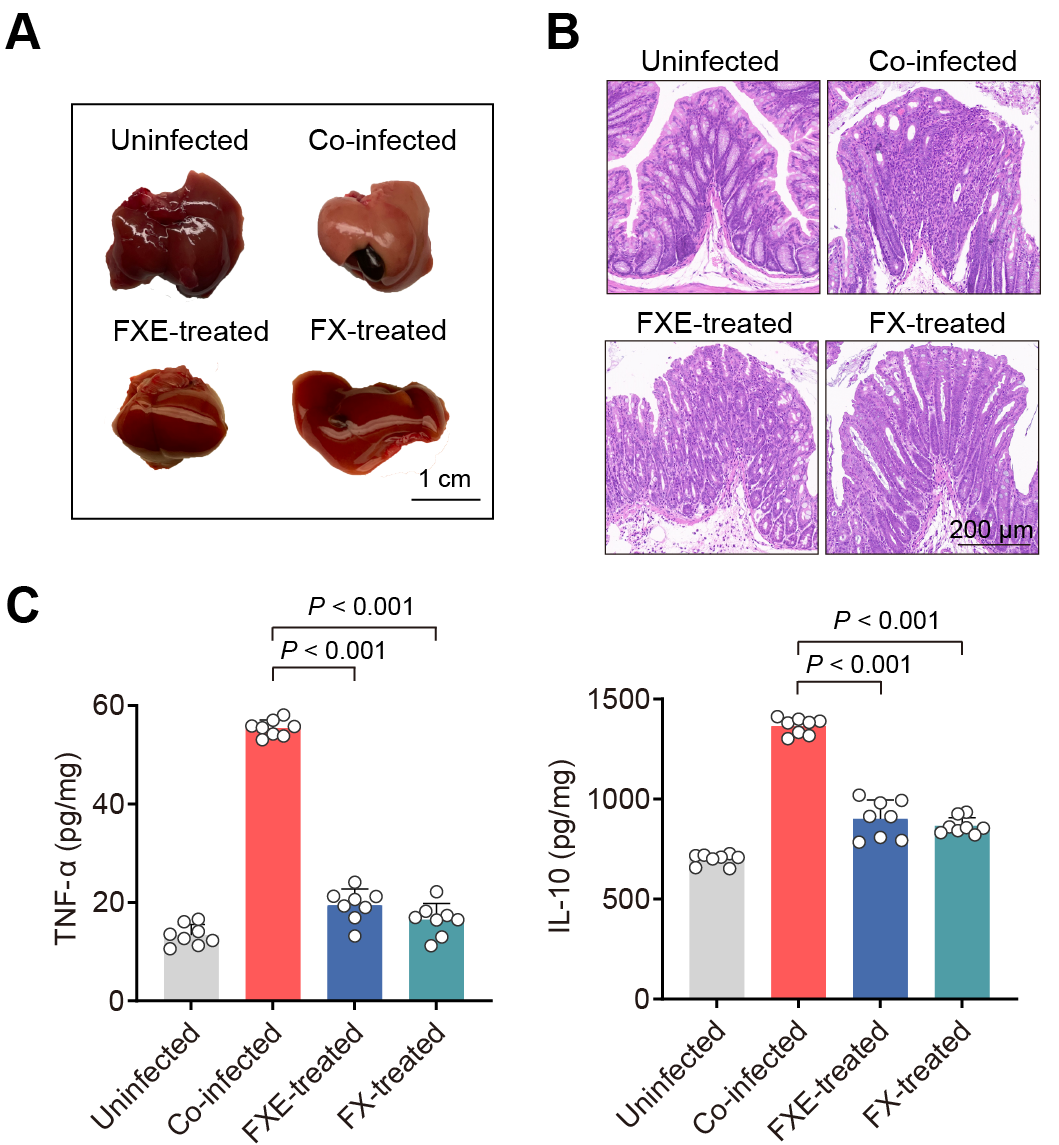

Supplement: Supplementary 1 — Figs. S1 to S8 Tables S1 to S5 [file research.0374.f1.zip › Figure S8.tif]
